# Integrated Analysis of Small RNA, Transcriptome, and Degradome Sequencing Reveals the MiR156, MiR5488 and MiR399 Are Involved in the Regulation of Male Sterility in PTGMS Rice

**DOI:** 10.3390/ijms22052260

**Published:** 2021-02-24

**Authors:** Yujun Sun, Xinguo Xiong, Qian Wang, Lan Zhu, Lei Wang, Ying He, Hanlai Zeng

**Affiliations:** MOA Key Laboratory of Crop Ecophysiology and Farming System in the Middle Reaches of the Yangtze River, College of Plant Science and Technology, Huazhong Agricultural University, Wuhan 430070, China; yujunsun@webmail.hzau.edu.cn (Y.S.); 18170844334@163.com (X.X.); samwang@webmail.hzau.edu.cn (Q.W.); lanzhu@mail.hzau.edu.cn (L.Z.); loudiwanglei@163.com (L.W.)

**Keywords:** PTGMS, male sterile, MicroRNAs, transcriptome, degradome

## Abstract

A photoperiod- and thermo-sensitive genic male sterile (PTGMS) line is the basic material for two-hybrid rice and is an important genetic breeding resource. Peiai64S (PA64S) is an important germplasm resource of PTGMS rice, and it has been applied to two-line hybrid rice systems in China. Pollen fertility in PA64S is regulated by the temperature and photoperiod, but the mechanism of the fertility transition is unclear. In this study, we obtained the male fertile plant PA64S(F) and the male sterile plant PA64S(S) by controlling different temperatures under long light conditions and used the male fertile and sterile plants to investigate the role of microRNAs (miRNAs) in regulating male fertility in rice. We performed the small RNA library sequencing of anthers from PA64S(S) and PA64S(F). A total of 196 miRNAs were identified—166 known miRNAs among 27 miRNA families and 30 novel miRNAs. In the transcriptome analysis, the Gene Ontology (GO) and Kyoto Encyclopedia of Genes and Genomes (KEGG) pathway analysis of differentially expressed genes revealed significant enrichment in the synthesis and metabolism of fatty acids and some secondary metabolism pathways such as fatty acid metabolism and phenylalanine metabolism. With a comprehensive analysis of miRNA, transcriptome, and degradome sequencing, we identified that 13 pairs of miRNA/target genes regulated male fertility in rice by responding to temperature change, among which the miR156, miR5488, and miR399 affect the male fertility of PA64S by influencing SPLs, the lignin synthesis of anther walls, and the flavonoid metabolism pathway. The results provide a new understanding of PTGMS rice, which will help us better understand the potential regulatory mechanisms of male sterility in the future.

## 1. Introduction

Rice is one of the main cereal crops in China and the world, providing a food source for nearly 50% of the global population. With the reduction in available arable land and the increase in the global population, it is estimated that by 2050, global food will need to increase by 70% to meet demand (FAO data). As the world’s largest rice producer and consumer, hybrid rice breeding has greatly increased food production in recent decades and made outstanding contributions to global food security [1]. Since the 1970s, hybrid rice production has mainly involved three-line and two-line hybrid systems [2]. The three-line hybrid system is based on male sterile lines, restorer lines, and maintainer lines, which have higher requirements for germplasm resources [3,4]. The two-line hybrid system method is based on an environmentally sensitive genic male sterile line; it uses a photoperiod- and thermo-sensitive genic male sterile (PTGMS) line to produce hybrid seeds, thus eliminating the need for maintainer lines and strict restorer–maintainer relationship restrictions, which makes it more conducive to superior hybrid configuration and hybrid seed production [5,6,7].

The utilization of the PTGMS line has become an important approach to promote the development of two-line hybrid rice system breeding [8]. The first natural mutant of PGMS rice, NK58S, was discovered in 1973 by the breeder Mingsong Shi in the field of Nongken 58 (*Japonica* rice) in Hubei Province, China [9]. The fertility of NK58S was observed to be regulated by day length, and NK58S is fertile in the short-day season but sterile in the long-day season. The fertility of rice was further found to be controlled by both day length and temperature. In 1986, the first TGMS rice mutant, 5460S, was discovered from the cytoplasmic male sterile recovery line 5460. The mutant 5460S became fertile at a low temperature but showed different degrees of complete sterility at a high temperature [10]. After years of screening and identification by breeders, PGMS and TGMS varieties such as Mian 9S, Yi D1S, Annong S-1, Zhu 1S, TGMS-VN1 and Sokcho-MS have been identified [11,12,13,14,15,16]. More studies have been conducted on the genetic analysis of different PTGMS rice varieties. Zhang located two loci, PMS1 and PMS2, on chromosomes 3 and 7, respectively, in the PGMS progeny of NK58S and found that the role of PMS1 was much greater than the role of PMS2 [17]. A large number of PTGMS rice candidate genes such as *tms*1, *tms*2, *ptgms*2-1, and *p/tms*12-1 were subsequently discovered by scholars [18,19,20,21]. These genes are distributed on the 11 chromosomes in rice, and there has been speculation that different candidate genes on the same chromosome may be different mutants of the same gene [22].

MicroRNAs (miRNAs) are endogenous noncoding small RNAs (sRNAs), typically with approximately 21–24 nucleotides, that can regulate gene expression by cutting mRNA or inhibiting translation [23]. miRNAs are involved in the regulation of various biological processes, including organ development, hormone signaling, and biotic or abiotic stress responses [24,25,26,27,28,29,30]. An increasing number of studies have identified a role for miRNAs in regulating plant male sterility. Vahid Omidvar et al. performed small RNA library sequencing of the PGMS mutant tomato 7B-1 with a wild-type reference. Thirty-two known miRNAs and 23 novel miRNAs were identified, and the expression levels of most miRNA/target pairs were found to be negatively correlated [31]. Zhang et al. performed small RNA and transcriptome sequencing of cytoplasmic male sterile cotton buds and found that gra-miR7505b could precisely cut 643 and 748 nt sites to regulate the pentatricopeptide repeat (PPR; Gh_D05G3392) gene, leading to the upregulation of the PPR gene and the restoration of male fertility [32]. Other studies have also found that miRNAs regulate gene expression, mostly through the cutting of target genes to affect anther and fertility development [33,34]. The miRNAs identified in cotton anther development after high-temperature stress were divided into seven categories according to miRNA families, and Chen et al. found that the miRNA responses to high-temperature stress were specific to anther changes at different developmental stages [35]. Recently, miRNA expression profiles of rice anthers in PTGMS under high and low temperatures have also been reported, thus providing a beneficial pathway for understanding the role of miRNAs in rice anther and PTGMS development.

Many miRNAs associated with anther development have been identified in crops. However, there is still a gap in the regulation of the fertility of the PTGMS rice line by miRNA and target genes. More research is needed to explain the molecular mechanism of the male fertility transition. Consequently, we obtained different fertile plants of Peiai64s (PA64S) by controlling the temperature under long light conditions. Sterile (S) and fertile (F) plants were constructed as PA64S sequencing libraries, and a number of known and novel miRNAs were identified. Degradome sequencing and target gene prediction were performed, and the miRNA and mRNA expression profiles of young spikes of fertile and sterile plants were comprehensively analyzed. This study provides a valuable resource for genome-wide studies of PTGMS rice line fertility-related genes and the mechanism of miRNA-mediated fertility transition.

## 2. Results

### 2.1. Abnormal Anther Development in PA64S under High-Temperature Treatment

**Figure 1:**
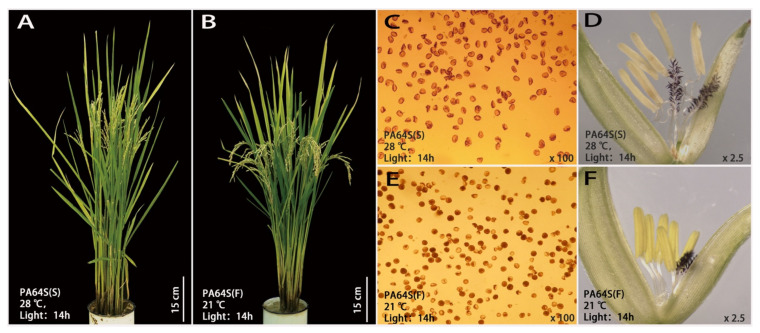
Plant morphology and characteristics of the anthers and pollen in sterile Peiai64s (PA64S(S)) and fertile Peiai64s (PA64S(F)). (**A**,**C**,**D**): pictures of plant morphology, pollen staining, and spikelets in PA64S(S), respectively; (**B**,**E**,**F**): pictures of plant morphology, pollen staining and spikelets in PA64S(F), respectively.

PA64S is a sterile line with excellent stability and genetic purity, and it exhibits fertility below 23 °C. PA64S(S) showed complete abortion and nonstainable pollen at high temperature (Figure 1A), and pollen grains were mostly triangular or nonrounded. While PA64S(F) pollen showed partial fertility and partial staining at a low temperature (Figure 1B), all the pollen grains were circular. Table 1 shows the pollen fertility and seed setting rate of PA64S(S) and PA64S(F) during 2018–2019. The average pollen iodine staining rate and seed setting rate of PA64S(F) were higher than 30% in both years, and the morphology of the plant panicle displayed a slight vertical downward trend. The results illustrated that PA64S male sterility can be partially restored to fertility under a low temperature and produces fertile plants that can fill and bear seeds normally. There was a significant difference in organ structure between PA64S(S) and PA64S(F). PA64S(S) was deficient in the development of some anthers, and the surface of the anthers was curved and sunken, the filament length was uneven, the organ was thin overall, and the anther color was nearly primrose yellow (Figure 1D). The PA64S(F) anthers developed normally. All six anthers had the same growth and a smooth surface, all filaments had the same thick and strong length, the organ was robustly structured, and the anther color was nearly yellow (Figure 1F).

### 2.2. Expression Changes of miRNAs Are Involved in the Response to Temperature Variation in PA64S

**Figure 2:**
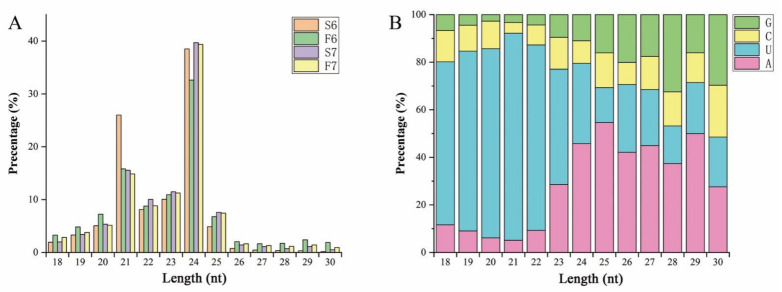
Lengths of small RNAs (sRNAs) and first bases of mature microRNAs (miRNAs) in sequencing libraries. (**A**) Size distribution of sRNA sequences identified in the sequencing libraries. The X-axis represents sRNAs of different lengths, while the Y-axis displays the number of sRNAs of a certain length. (**B**) The first base preference of mature miRNAs in the sequencing libraries. The X-axis represents the length classification of the sRNAs, and the Y-axis displays the proportion of mature miRNAs with a certain base type as the first base.

To investigate the role of miRNAs in response to temperature variation in PA64S, the sRNA libraries were constructed and sequenced using Illumina sequencing platform technology. The correlation coefficients of the samples are shown in Supplementary Figure S1. In total, 60 million raw reads were obtained from the sequencing libraries, and each sample read remained above 15 million (Table S1). After removing raw reads containing spliced and low-quality sequences, 62,076,461 clean reads were obtained. sRNAs within a certain length range were selected for the subsequent analysis of sRNA distribution, and sRNAs of 20–25 nt in length were dominant in all libraries, accounting for 92.65%, 89.71%, 82.18% and 86.90% of all clean reads in the S6, F6, S7 and F7 libraries, respectively (Figure 2A). Among these sRNAs, the most abundant was 24 nt sRNA (all above 32%), followed by 21 nt sRNA (between 10.68% and 26.00%). These results were consistent with the results concerning rice anthers. Mature 20–23 nt miRNAs mostly started with ‘U’ as the first base (48.49–87.07%), while 24 nt miRNAs mostly started with ‘A’ (45.80%) (Figure 2B). These total clean reads contained miRNA, rRNA, snRNA, tRNA, snoRNA, and unannotated sequences (Table S2). More than 80% of these clean reads could be compared to a reference sequence to identify miRNAs.

**Figure 3:**
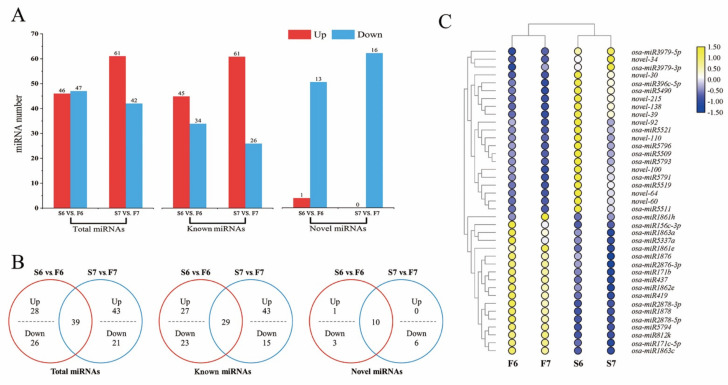
Differentially expressed miRNAs in PA64S(S) and PA64S(F). (**A**) The number of miRNAs up- or downregulated in stages 6 and 7. (*p* < 0.05) (**B**) Venn diagrams showing the unique and shared differentially expressed miRNAs in stages 6 and 7. (**C**) Hierarchical cluster analysis of 39 differentially expressed miRNAs in stages 6 and 7. The relative expression levels in the figure are the average of three replications.

We identified 196 miRNAs in two stages, 166 (84.7%) of which were known miRNAs and 30 of which were novel miRNAs (Table S3). Of the 196 miRNAs, 53 miRNAs in stage 6 and 103 miRNAs in stage 7 were considered to be differentially expressed miRNAs in response to temperature variations (Figure 3A). Compared with PA64S(F) and PA64S(s), 107 miRNAs (including 46 of stage 6 and 61 of stage 7) were upregulated, and 89 miRNAs (including 47 of stage 6 and 42 of stage 7) were downregulated. The analysis of differentially expressed miRNAs in stages 6 and 7 revealed that 39 miRNAs exhibited a response to temperature changes in both stages, including 29 known miRNAs and 10 novel miRNAs (Figure 3B). Figure 3C shows the expression profiles of miRNAs that responded to temperature variations at two stages. Among these 39 miRNAs, 21 miRNAs showed an upregulated expression in S6, and 18 miRNAs showed a downregulated expression in S6; however, most of the miRNAs responding to the temperature change in stage 7 were downregulated. The secondary structure of the novel miRNAs was shown in Supplementary File 1.

A total of 27 miRNA families were identified from the differentially expressed miRNAs in the libraries (Table 2), including 15 miRNA families (including 20 miRNAs) and 21 miRNA families (including 34 miRNAs) differentially expressed in stages 6 and 7, respectively. Among these families, nine miRNA families were expressed in both stages: MIR171-1, MIR1861, MIR1862, MIR1863, MIR1878, MIR2118, MIR2863, MIR437 and MIR812. Except for MIR812, all of these genes were upregulated. In addition, six miRNA families were unique in stage 6; four of the miRNA families were upregulated, and two miRNA families were downregulated. A total of 12 miRNA families were unique in stage 7; seven of the miRNA families were upregulated, and four miRNA families were downregulated. However, two miRNAs in the MIR2275 family showed upregulated and downregulated expression trends.

**Figure 4:**
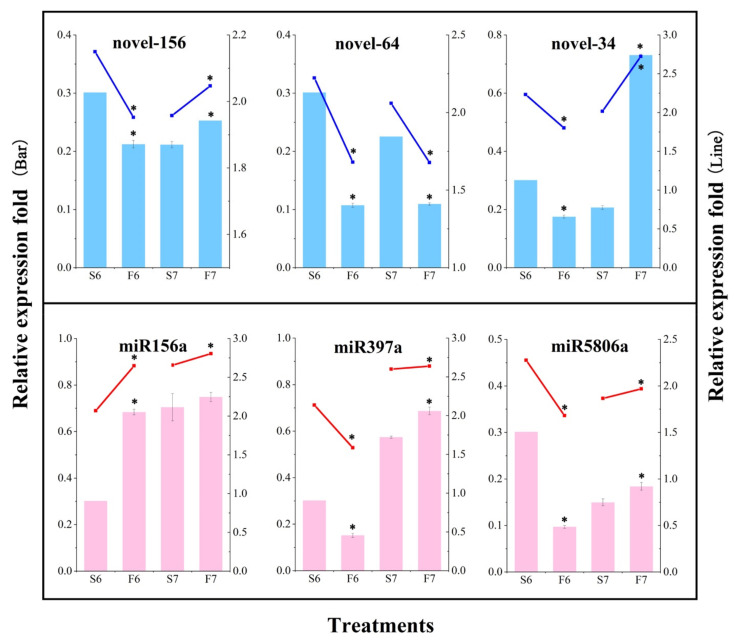
Validation of the relative expression level of partial known and novel miRNAs by sRNA sequencing and qPCR in PA64S(S) and PA64S(F). Dark blue lines indicate relative changes in the expression levels of three differentially expressed novel miRNAs, as determined by RNA-Seq. Blue bars indicate relative changes in the expression levels of the novel miRNAs, as determined by qPCR. Red lines indicate relative changes in the expression levels of three differentially expressed known miRNAs, as determined by RNA-Seq. Pink bars indicate relative changes in the expression levels of the known miRNAs, as determined by qPCR. The average values of three biological repeats are shown. Error bars represent standard deviations. Asterisks indicate significant differences revealed by Student’s *t*-test at *p* < 0.05 (*).

To verify the accuracy of the miRNA sequencing data, six differentially expressed miRNAs were randomly selected for qPCR analysis (Figure 4). Three known miRNAs and three novel miRNAs were randomly selected from the anthers of PA64S(S) and PA64S(F), and their expression was determined in stages 6 and 7. Novel-64 showed a higher expression in sterile plants than fertile plants in both stages 6 and 7, whereas the known miR156a showed a higher expression in fertile plants than in sterile plants in both periods. The expression trends of each miRNA measured by qPCR were consistent with the expression trends of sRNA sequencing results, indicating that the sRNA sequencing results were reliable.

### 2.3. Changes in Gene Transcript Levels Are Involved in the Response to Temperature Variation in PA64S

To investigate changes in target gene expression in response to temperature variation in PA64S, transcriptome libraries were constructed using the same samples used for the miRNA sequencing (Table S4). A total of 846,119,950 (60–82 million) raw reads were generated by transcriptome sequencing, averaging 70 million reads per library. Ninety percent of these reads successfully mapped to the reference genome, and approximately 1.56% of the reads successfully mapped to multiple regions.

**Figure 5:**
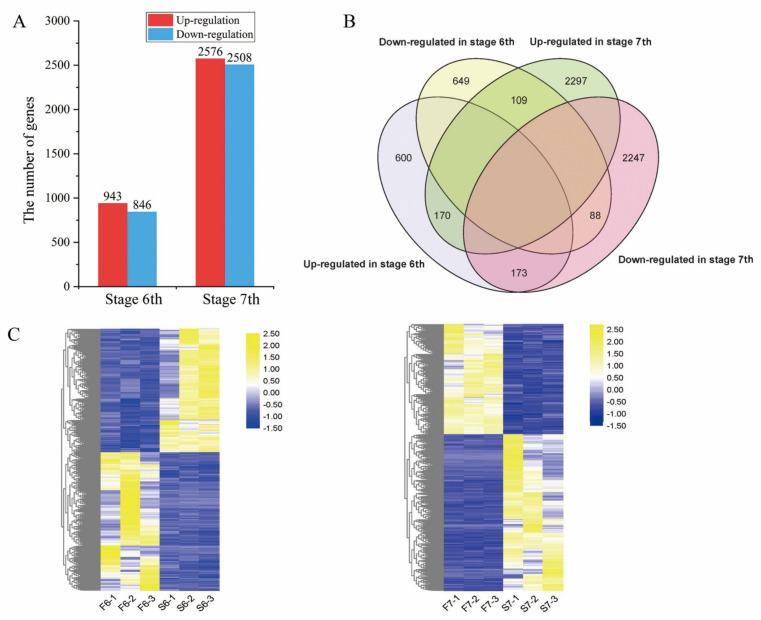
Differentially expressed genes (DEGs) in PA64S(S) and PA64S(F). (**A**) The number of genes upregulated or downregulated in response to temperature by > 1-fold in stages 6 and 7 (*p* < 0.05). (**B**) Venn diagram showing the unique and shared DEGs in stages 6 and 7. (**C**) Hierarchical cluster analysis of DEGs in stages 6 and 7.

Global gene expression in PA64S was further investigated. We used padj < 0.05 combined with |fold change| > 1 to select significantly differentially expressed genes (DEGs) between PA64S(F) and PA64S(S) at the same stage (Table S5). Overall, a total of 1789 DEGs were identified in PA64S(S) compared to PA64S(F) in stage 6, of which 943 were upregulated and 846 were downregulated. A total of 5084 DEGs were identified in stage 7 in PA64S(S) compared with PA64S(F), of which 2576 showed an upregulated expression and 2508 showed a downregulated expression (Figure 5A). We found 540 genes differentially expressed in both stages 6 and 7 (Figure 5B), of which 170 genes were upregulated and 88 were downregulated in both periods. The mRNA expression patterns of stages 6 and 7 are shown in Figure 5C, and the results indicated that the expression of the DEGs was mostly downregulated in stage 6; in contrast, the expression of DEGs was mostly upregulated in stage 7.

**Figure 6:**
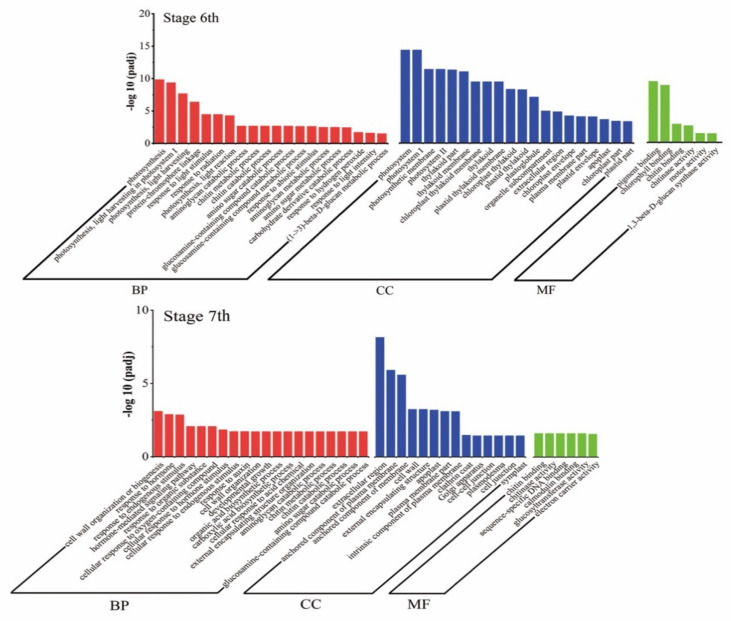
Gene Ontology (GO) analysis of DEGs in stages 6 and 7. Genes are classified into three major categories: biological process, cellular component, and molecular function. The X-axis indicates the −log10 (padj) of GO, and the Y-axis indicates the GO terms of genes.

The Gene Ontology (GO) analysis of DEGs was performed, and DEGs were classified into three major categories: biological processes, cellular components, and molecular function (Figure 6). At stages 6, 24, 33 and 6 GO terms were significantly enriched in biological processes, cellular components, and molecular function, respectively. At stages 7, 34, 14 and 6, GO terms were significantly enriched in biological processes, cellular components, and molecular function, respectively. Biological processes associated with “photosynthesis” and “photosynthesis light reaction” were significantly enriched in stages 6, and in 7, the main biological processes were enriched in “response to hormone”, “hormone-mediated signaling pathway”, and “cellular response to hormone stimulus”. Among the cellular components, “photosystem”, “photosynthetic membrane”, and “thylakoid part” were particularly prominent in stage 6, while the “extracellular region” and “component of plasma membrane” were prominently enriched in stage 7. Among molecular functions, both periods were prominent in “chlorophyll binding” and “chitin binding”.

**Figure 7:**
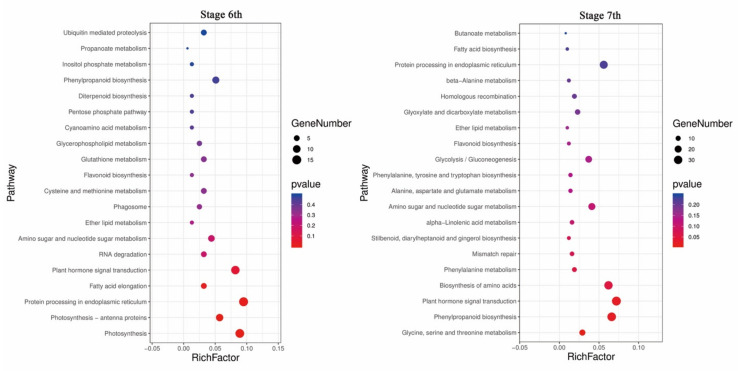
Kyoto Encyclopedia of Genes and Genomes (KEGG) enrichment analysis of DEGs in stages 6 and 7. The top 20 enriched pathways in stages 6 and 7 are shown. Each circle represents a KEGG pathway. The X-axis represents the enrichment factor, which compares the ratio of genes annotated to a pathway among the DEGs to the ratio of genes annotated to that pathway among all genes, and the Y-axis represents the pathway name.

To better understand gene function and regulatory networks, a Kyoto Encyclopedia of Genes and Genomes (KEGG) analysis of temperature differential genes was performed, and 69 and 98 pathways were enriched in stages 6 and 7, respectively. We identified the top 20 enriched pathways, which are summarized in Figure 7, and the main enriched pathways were “photosynthesis”, “plant hormone signal transduction”, and “biosynthesis and metabolism of amino acids and fatty acids”. Among these pathways, the “plant hormone signal transduction” pathway was significantly enriched, indicating that the hormone signal was involved in the changes in rice fertility and played a key role in fertility regulation. The “fatty acid synthesis”, “ether lipid metabolism”, and “sphingolipid metabolism” pathways were also enriched at the same time in both stages, and it has recently been published that fatty acid metabolism is involved in the regulation of fertility in PTGMS rice [36]. Interestingly, the synthesis and metabolism of fatty acid and amino acid pathways, such as fatty acid biosynthesis and metabolism and phenylalanine biosynthesis and metabolism, were concurrently enriched. In addition, some secondary pathways such as “flavonoid biosynthesis” and “diterpenoid biosynthesis” were also enriched.

### 2.4. Target Identification of miRNAs by Degradome Analysis

**Figure 8:**
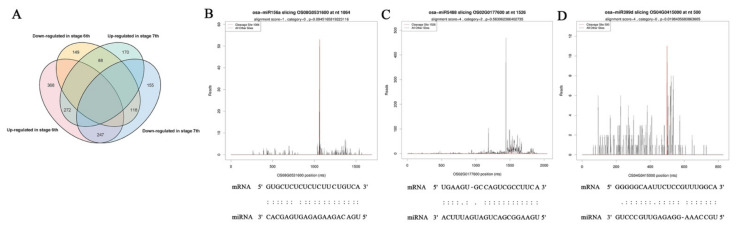
Analysis of known miRNA targets by degradome sequencing and statistics for miRNA targets. **(A**) Venn diagram of differentially expressed target genes in stages 6 and 7 of PA64S(S) and PA64S(F). (**B**) Targets of osa-miR156a. (**C**) Targets of osa-miR5488. (**D**) Targets of osa-miR399d. Red lines indicate the signature produced by miRNA-directed cleavage.

To determine the regulatory relationship between miRNAs and their target genes, degradome libraries for the same samples were constructed. In the obtained raw data (Table S6), the reads of each library averaged 27 million (24,983,037–32,138,241), unique mappable reads were all greater than 99%, unique transcript mapped reads were stable at approximately 80%, the number of covered transcripts ranged from 52,925 to 58,864, and the results indicated that degradation group sequencing yielded a high coverage of degradation fragments. Combined with target gene prediction software, a total of 317 miRNAs and 1568 candidate target genes were identified (Table S7), of which 725 were stage 6 and seven shared target genes (Figure 8A). According to the signature number and abundance of putative cleaved positions at each occupied transcript, these cleaved transcripts could be categorized into five classes according to the signature abundance at each occupied transcript position: 0, 1, 2, 3 and 4. Types 0, 1, 2, 3 and 4 had 633, 99, 1440, 195 and 851 cleavage events, respectively (Table 3), and the target plot (t-plot) of four types of miRNA cutting target genes is shown in Supplementary Figure S2. The signature abundance of each target was plotted using an absolute read number along the target transcripts. The t-plots of known miRNA targets confirmed that degradome sequencing occurred during the rice male fertility transition. The alignment between representative miRNAs and their target transcripts are shown below the corresponding t-plot (Figure 8B–D). The results indicated that the results of the cutting between miRNA and target genes were accurate and reliable.

### 2.5. Comprehensive Analysis of miRNA Expression Profiles and Target Genes in Response to Temperature Variation in PA64S

To determine the role of miRNAs in response to temperature changes, we integrated combined sRNA, degradome, and transcriptome sequencing to analyze the expression profiles of miRNAs and target genes. Four and nine miRNA—target gene pairs were identified in stages 6 and 7 in response to fertility changes, respectively (Table 4). In these thirteen pairs, four pairs comprised one miRNA corresponding to multiple target genes, and nine miRNA—target pairs were expressed one-to-one. These one-to-one miRNA—target pairs included six upregulated miRNA/downregulated targets, two downregulated miRNA/upregulated targets, and one upregulated miRNA/upregulated target. Microspores with reproductive functions require pollen mother cells to be produced through mitosis and meiosis. Therefore, cell division plays an important role in pollen formation. Among the targets, a downregulated target of osa-miR156a, OS08g0531600, is a transcription factor in cell division-related processes. In the secondary metabolism process, the target gene OS01g0606000 (bidirectional sugar transporter) of miR419 and the target gene OS02g0177600 (4-coumarate-CoA ligase 3) of miR5488 and the target gene of miR399d, OS04g0415000 (a coding gene similar to the H0622F05.5 protein), were also significantly different. The results indicated that the glycan transport and lipid synthesis pathways are involved in the regulation of rice male fertility by responding to changes in temperature.

**Figure 9:**
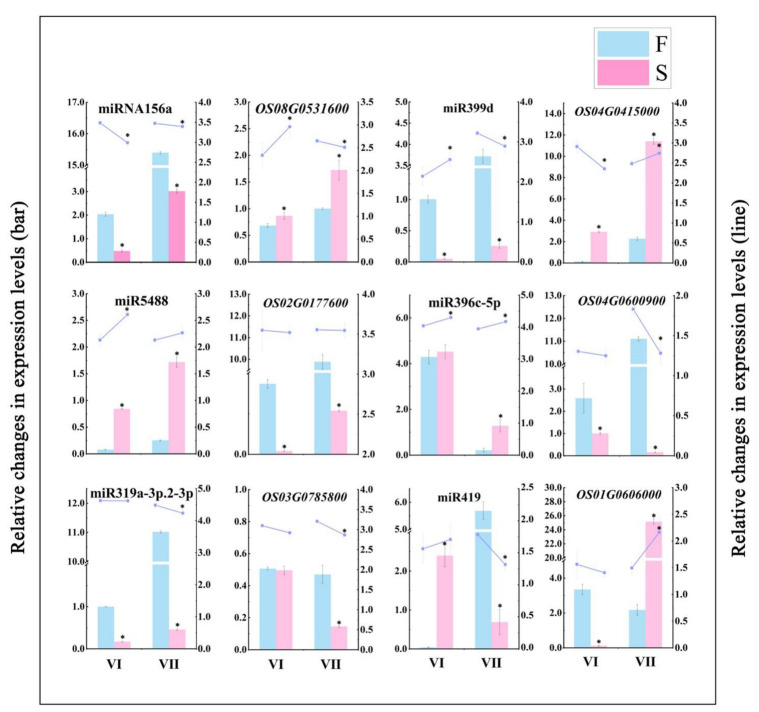
Validation of differentially expressed miRNAs and their corresponding target genes. Bars indicate relative changes in the expression levels of differentially expressed miRNAs and target genes, as determined by RNA-Seq. Lines indicate relative changes in the expression levels of differentially expressed miRNAs and target genes, as determined by qPCR. The average values of three biological repeats are shown. Error bars represent standard deviations. Asterisks indicate significant differences revealed by Student’s *t*-test at *p* < 0.05 (*).

To validate the sequencing and analysis process, the relative expression levels of the six miRNA—target gene pairs we identified were validated using qPCR (Figure 9). The results showed that the expression patterns shown by the qPCR analysis of miRNA—target gene pairs were similar to those produced by high-throughput sequencing, thus confirming that the sequencing results and the analysis process were authentic and reliable.

### 2.6. The Content of Metabolites and Relative Expression Level of Related Genes Involved in the Regulation of Male Fertility Processes

**Figure 10:**
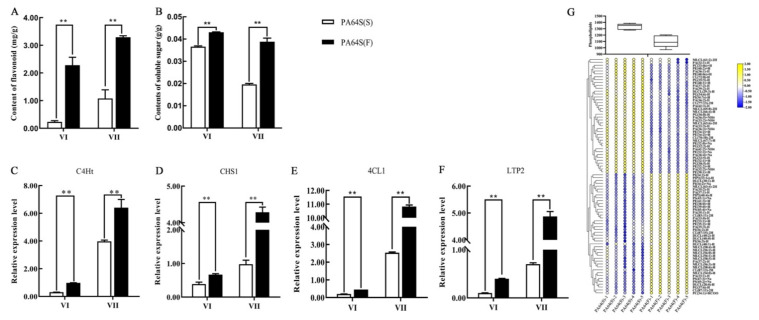
The content of metabolites and relative expression of related genes involved in the regulation of male fertility processes. (**A**) Content of flavonoid, (**B**) content of soluble sugar, (**C**) relative expression level of C4Ht, (**D**) relative expression level of CHS1, (**E**) relative expression level of 4CL, (**F**) relative expression level of LTP2, and (**G**) phospholipid content of phospholipid and different lipid groups in PA64S(S) and PA64S(F) lipid metabolism. The box plot indicates the total content of phospholipids. CL: cardiolipin; DLCL: di-lyso cardiolipin; MLCL: mono-lyso cardiolipin; PA: phosphatidic acid; PC: phosphatidylcholine; PE: phosphatidylethanolamine; PG: phosphatidylglycerol; PI: phosphatidylinositol; PIP2: phosphatidylinositol diphosphate; and PS: phosphatidylserine. Asterisks indicate significant differences revealed by Student’s *t*-test at *p* < 0.01 (**).

To better demonstrate the involvement of our predicted miRNAs and their target genes in the rice male fertility transition process, we measured the content of some metabolites and the expression levels of related genes in the regulatory pathways in which some of these miRNAs and their target genes may be involved (Figure 10). Flavonoid metabolism is an important branch of phenylpropanoid metabolism and gives rise to the largest class of polyphenolic metabolites. From the content of flavonoids, the main product of secondary metabolism, the content was higher in PA64S(F) in two stages (Figure 10A), which indicated the more active metabolism and enrichment of flavonoids in the young spikelets of fertile plants. The same conclusion could be reached in the expression of *CHS1*, the main gene in flavonoid synthesis (Figure 10D). Phenylpropanoid metabolism is an upstream pathway of flavonoid metabolism, and *C4Ht* is an important metabolic rate-limiting gene. The relative expression level of *C4Ht* was greater in PA64S(F) than in PA64S(S) in two stages of PA64S (Figure 10C), and the results indicated that the general phenylpropanoid pathway was more active in fertile plants. Meanwhile, we quantified the *4CL1* gene common to the general phenylpropanoid pathway and the downstream lignin synthesis pathway and found that its expression trend was the same as that of *C4Ht* (Figure 10E). The results of *C4Ht*, *CHS1*, and *4CL1* indicated that the general phenylpropanoid pathway and its two downstream flavonoid and lignin pathways are more active in PA64S(F) plants. Finally, differences in soluble sugar content and phospholipid metabolism levels were determined in the PA64S(F) and PA64S(S) plants. The content of soluble sugar showed a higher level in fertile plants in two stages (Figure 10B), while the total phospholipid content showed a lower level in fertile plants (Figure 10G). The difference in the contents of phospholipid between the different groups showed that there were fewer groups exhibiting downregulation in PA64S(F) compared to PA64S(S), and the large fold change resulted in a decrease in the total phospholipid content. Additionally, we found that the activity of the lipid transporter gene *LTP2* was significantly differentially expressed in the sterile and fertile plants, and the results indicated the important role of lipid metabolic processes in fertility transition.

## 3. Discussion

### 3.1. Abnormal Tapetum Causes Male Sterility under High Temperature in PA64S

The mechanism of fertility transformation and the selection of new sterile lines of photothermally sensitive male sterile rice have been the focus of attention as the basis of two-hybrid rice. The PA64S plants used in this study were PTGMS rice varieties selected by ten generations of continuous directional temperature and agronomic traits that are relatively stable in terms of growth stages and fecundity critical temperature characteristics [37]. The control of fertility in male plants is essential for plant reproduction and selective breeding, and it involves multiple steps in stamen development [38]. The abnormal degradation of the anther tapetum, defective anther wall development, and abnormal anther dehiscence have been found to be the main factors affecting male sterility in rice [39,40,41]. In our study, the fertility of pollen between PA64S(S) and PA64S(F) was significantly different. PA64S(S) anthers were more thin and yellowish compared to PA64S(F), which is consistent with the anther phenotype observed by Song et al., who found that the anthers of the male sterile mutant of *Osttip7* were not completely dehiscent, thus failing to release mature pollen grains and eventually leading to sterility [42]. However, anther closure and dehiscence abnormalities did not occur in our experimental material, so there has been speculation that PA64S(S) anther sterility has another cause. Research on the cytological aspects of rice pollen by electron microscopy is well-established, and other studies have found differences between male sterile rice pollen and normal fertile pollen [43]. Recent studies have found that the male sterile mutant BM1 showed abnormalities in male meiosis, tapetal development, and degeneration timing, as well as pollen exine formation, ultimately leading to microspore degeneration [44]. In our previous research, the results showed that the mature pollen grains in PA64S(F) were subglobose-shaped, and there were regularly arranged sporopollenin on the surface of the pollen wall [36]. However, the pollen grains in PA64S(S) were severely shrunken, and sporopollenin was loosely distributed on the pollen wall. Compared with PA64S(F), the tapetum of PA64S(S) was not degraded. Xiang et al. [44] also confirmed our results, and this phenomenon indicated that the abnormal development of the tapetum is the main factor responsible for male sterility in rice. Therefore, we inferred that the delayed degradation of the tapetum, which resulted in uneven distribution of sporopollenin on the surface of the pollen wall, might be responsible for the sterility of PA64S(S) pollen.

### 3.2. MiRNAs Are Involved in the Regulation of Male Fertility in PA64S in Response to Temperature Changes

miRNAs are widely distributed endogenous sRNAs ranging from 20 to 24 nucleotides in length. miRNA inhibits translation or guides the degradation of its mRNA target and is a key regulator of posttranscriptional gene regulation [23,45]. Several studies have shown that anther development is a key biological process for sexual reproduction in plants, and miRNAs play a key role in regulating plant anther development [46,47]. In this study, the abundance of 21 and 24 nt sRNAs was found to account for a relatively high proportion in the essential stages for the fertility transition of anther development in PA64S. We also drew similar conclusions by comparing the distribution of sRNA richness in other plants such as peanuts, rapeseed, tomatoes, and radishes. However, we found that 18 nt sRNAs have the highest richness in wheat [31,33,48,49,50]. Such results indicated that the abundance of sRNAs may only slightly vary between species, regardless of experimental treatment and variety selection.

MiRNAome and degradation sequencing technologies have become effective methods to identify and evaluate the expression profiles of miRNAs and related targets in plant tissues [47,50]. Our results illustrated that changes in the expression of miRNAs play a major role in the response to temperature changes, but there was no significant difference in the number of total miRNAs, known miRNAs, and new miRNAs identified in the two stages. Therefore, we can infer that miRNA responds to temperature changes and participates in the process of fertility changes by regulating the upregulation or downregulation of miRNA expression in cells. The miRNA expression profiles shared in PA64S(S) and PA64S(F) revealed that the expression of the same miRNA changes regularly with the change in fertility difference materials, and the same miRNA shows different expression patterns between PA64S(S) and PA64S(F). This result was also consistent with the conclusion of Nie et al. that miRNA changes during the development of CMS cotton flower buds [51]. In this study, 54 miRNAs, including MIR156, MIR164, MIR169, MIR395, MIR399, and MIR820, were classified as known miRNA families (Table 2). These miRNA families are conserved in most plant species, and some are conserved across the huge evolutionary distances between mosses and flowering plants [52]. Conserved miRNAs have homologous target mRNAs in multiple species, so the miRNA–target relationship is usually stable over a long period in plant evolution [53,54]. Other studies have shown that the MIR159 and MIR169 families play an important regulatory role in the transitional stage of plant reproduction [55,56]. Ding et al. found that high temperature inhibits the expression level of miR156, thereby changing the expression of its targeted transcription factor, squamosa promoter binding protein-box gene (*SPL*), disrupting the development of floral organs and leading to male sterility in cotton [57]. Similarly, miR156 has also proven to be the target gene of *SPL*, which can regulate cell division and differentiation in the early stage of anther development and plays an important role in the change in anther fertility [58,59]. The results indicated that miR156 is responsive to temperature changes and involved in regulating male fecundity. Sun et al. used multiomics comprehensive analysis and found that the expression of key miRNAs related to anther development changed during the development of light-heat-sensitive male sterile wheat, especially tae-miR1127a. Its target gene SMARCA3L3 may be related to the development of meiosis in male germ cells. The male fertility of 337S is believed to be the result of tae-miR2275-CAF1 and tae-miR1127a-SMARCA3L3 miRNA—target coregulation [60]. Recently, the role of the MIR399 family in regulating reproductive development and male fertility in plants was reported [61]. Wang et al. found that miR399a has the highest abundance in floral organs of citrus and verified that CsUBC24 (PHO2) is the target gene for the miR399a-mediated suppression of expression. In addition, CsUBC24 interacts with the SEPALLATA MADS box transcription factor family (CsSEP1.1, CsSEP1.2, and CsSEP3), which regulates flower development, and the transcription factor ICE1, which regulates stomatal development and affects the expression of the key gene CsLMI2 for flower development and anther dehiscence, thus proving that miR399 is closely related to floral organ development and pollen fertility. We therefore infer that miRNAs and the miRNA family all play an important role in the regulation of plant fertility.

### 3.3. MIR156, MIR5488, and MIR399 Regulated Male Fertility in Rice by Responding to Temperature Change

Many researchers have used high-throughput sequencing technology to screen a large number of potential miRNAs related to plant fertility changes. These miRNAs participate in important regulatory processes such as plant ovule development, flower bud differentiation, and anther development [62,63,64]. However, there have been few reports on the study of miRNAs involved in PTGMS rice fertility regulation. Plant genomics studies have shown that many miRNAs and their target genes are involved in the coordination of flower differentiation and development, and most miRNAs negatively regulate certain genes in plant development by cutting or inhibiting the translation of target transcripts [65]. Therefore, the accurate identification of differential miRNA and functional target gene expression during fertility development has become an effective way to find and regulate fertility genes.

The SPL transcription factor family is an important regulator of the entire growth and development stages of plants. MiR156-SPL modules have been reported to regulate multiple biological processes, including juvenile-to-adult phase transition; the development of leaves, roots, and fruit; fertility; stress response, and secondary metabolism [66,67,68]. In rice, miR156 targets SPL proteins, which play important roles in the proper development of sporogenic tissues. Previous studies have reported that miR156 regulates the timing of flower formation through its target *SPL3*, which activates the expression of APETALA1 [69]. *SPL3*, *SPL6*, and *SPL9* were identified as potential targets of miR156 family members, and there were threefold differences in different fertility treatments, leading to disorders of flower organ development in peppers, which proved that miR156 may be involved in plant fertility regulation [70]. Studies have found that there are multiple *SPL* genes in *Arabidopsis* that regulate cell division and differentiation and can produce fertile pollen. Xing et. al. [71] performed an analysis of the SPL gene family and revealed a large number of SPL gene family members targeted by miR156 in the unrooted phylogram of all *SPL* genes, as well as that SPL transcription factors act in concert to secure male fertility in *Arabidopsis*.

In the present study, the functional analysis of the identified target genes revealed that miR156a was downregulated in PA64S(S), and the target *SPL16* gene (OS08g0531600) was upregulated. Scholars have found that low expression levels of miR156 in sterile plants may affect anther formation during pollen development, and this conclusion is consistent with our findings [72]. Furthermore, an elevated ambient temperature also was found to cause increased miR156 expressions and the downregulation of miR156-targeted *SPL* [73], the results indicating that the ambient temperature is also an input signal for miR156-regulated pathways. Clearly, this result was contrary to our study, suggesting that the downregulation of miR156 may be in response to changes in fertility rather than in response to changes in temperature. In our past study, we found that the GA content differed significantly under different fertility conditions. The GA content was significantly higher in PA64S(F) than in PA64S(S) in the later stages of spike development, and the same trend was observed for the positive GA effector *GAMYB*. The promotion of flowering by GA in *Arabidopsis* was demonstrated to involve an interaction between DELLA proteins and SPLs, and several DELLA proteins interact and repress SPL protein activities [74]. We conjecture that GA may play a regulatory role between DELLA and SPL, participating with miR156 in regulating changes in SPLs. Therefore, we speculate that the significantly differentially expressed miR156a and its target genes identified in PA64S are involved in its fertility regulation process. In the subsequent qPCR verification (Figure 9), the expression pattern of miRNA—target gene pairs was also found to conform to our results.

Flavonoids are secondary metabolites that have antioxidant effects in higher plants. Flavonoids are biosynthesized by the shikimic acid and phenylpropanoid metabolic pathways, in which 4-coumarate-CoA ligase (4CL) catalyzes the last step of the phenylpropanoid metabolic pathway to produce coumaroyl-CoA, which synthesizes a variety of flavonoid products through a series of enzymatic reactions [75]. Flavonoids play an important role in the formation of pollen grains and are an important component of the outer wall of pollen. The outer wall absorbs flavonoids, carotenoids, lipids, proteins, and other substances synthesized during the disintegration of the tapetum for the synthesis of the pollen outer wall. The content of flavonoids was higher in PA64S(F) in two stages (Figure 10A), and the same conclusion could be reached for the expression of *CHS1*, the main gene in flavonoid synthesis. The results indicated that there was more active metabolism and enrichment of flavonoids in the young spikelets of fertile plants and that flavonoid metabolism is involved in male fertility transition. The cinnamic acid produced by the phenylpropanoid metabolic pathway is converted to lignin monomers by a series of enzyme-catalyzed reactions, with key enzymes including 4CL, caffeic acid O-methyltransferase (COMT), and caffeoyl-CoA O-methyltransferase (CCoAOMT). The enzyme 4CL is rate-limiting in the lignin monomer synthesis pathway, and it has been shown that the transgenic *Arabidopsis thaliana*, tobacco, and poplar that inhibit *4CL* expression have significantly reduced lignin contents [76,77,78]. The inhibition of *4CL* expression in rice decreases pollen fertility, possibly due to the disruption of lignin synthesis during pollen wall development [79]. In this study, the target gene of miR5488 was found to be *4CL-3* (OS02G0177600), a member of the important enzyme 4CL protein family in the flavonoid metabolism pathway and the lignin synthesis pathway. The upregulation of miR5488 in PA64S(S) resulted in the downregulation of its target gene *4CL*, and the expression of the 4CL family member *4CL1* also showed low levels in sterile plants. The results confirmed that male fertility is closely related to the metabolism of phenylpropane in which 4CL is involved. In our previous studies, both *4CL* and *COMT* were found to be downregulated in the sterile strain of the photoperiod-sensitive rice material D52S. This result also confirmed our suspicion that the inhibition of upstream *4CL* expression affects *COMT* expression, thereby blocking lignin synthesis and influencing pollen wall formation, which is involved in fertility regulation. Recent studies have also found that the phenylpropanoid pathway is essential for exine formation, and both sporopollenin and lignin share the same phenylpropanoid pathway [80]. In our research, numerous genes were found to be enriched in the phenylpropane metabolic pathway—which blocked the phenylpropanoid metabolic pathway, affecting flavonoid and lignin synthesis and producing sterility—in the KEGG analysis of DEGs, which confirmed our hypothesis.

In *Arabidopsis*, rice, and maize, the PHOSPHATE2 gene (*PHO2*) is targeted by miR399 [81,82,83], and *PHO2* negatively regulates inorganic phosphate (Pi) uptake and translocation by downregulating the expression of PSI-related genes [84]. Wang et al. also provided evidence that Pi uptake and translocation in citrus might be impeded by miR399a.1-STTM [61]. Alterations in lipid metabolism, resulting in phospholipid degradation and galactolipid and sulfolipid synthesis, could increase intracellular Pi availability under Pi depletion [85]. Meanwhile, genes related to phospholipid degradation, galactolipid synthesis, and sulfolipid synthesis were upregulated in rice roots upon Pi deprivation [86]. Pi was also found to regulate carbohydrate metabolism, and Pi deprivation in leaves was found to downregulate photosynthesis and induce increases in the levels of sugars and starch. Disturbed glucose metabolism significantly impairs pollen development and leads to male sterility [87,88].

Similarly, we found that the expression of the miR399 family is significantly different in PA64S(S) and PA64S(F) materials, the expression of miR399d is downregulated in PA64S(S), and its target gene OS04g0415000 coding the H0622F05.5 protein is upregulated. We did not find a function for the protein, but we found a homologous protein in *Arabidopsis* by sequence alignment, namely the homolog of the OS04g0415000 protein GL2 in *Arabidopsis*. *GL2* was found to negatively regulate anthocyanin biosynthesis in *Arabidopsis* by directly repressing the expression of some of the MYB-bHLH-WD40 activator complexes [89,90]. This finding is also similar to the conjecture that the miR5488 target gene *4CL* is involved in flavonoid metabolism to influence fertility. The flavonoid metabolic pathway has been proven to play an important role in the regulation of plant fertility, and multiple target genes may simultaneously regulate flavonoid metabolic pathways in rice to influence fertility changes. In the KEGG analysis of DEGs, we also found that the pathway of DEGs is also significantly enriched in the fatty acid metabolism and secondary metabolism pathways. The determination of phospholipids, an important component in lipid metabolism, revealed that the total phospholipid content was higher in sterile plants, while the relative expression of the transporter gene *LTP2* was lower (Figure 10). It has been found that the phospholipid-binding protein encoded by Ms1 transports phosphatidic acid (PA) and phosphatidylinositol (PI), and the proper localization of PA and PIs is critical for microgametogenesis in wheat for male fertility [91]. In our study, we found differences in PA and PI contents in PA64S(F) and PA64S(S) plants. PA content was upregulated in PA64S(F) among 11 groups, and PI was conversely upregulated in only one group (Figure 10G). Therefore, we speculate that miR399 regulates the relationship between lipid metabolism–Pi signaling–carbohydrate metabolism, as well as regulating the distribution of lipid and sugar substances, thus affecting growth and development.

**Figure 11:**
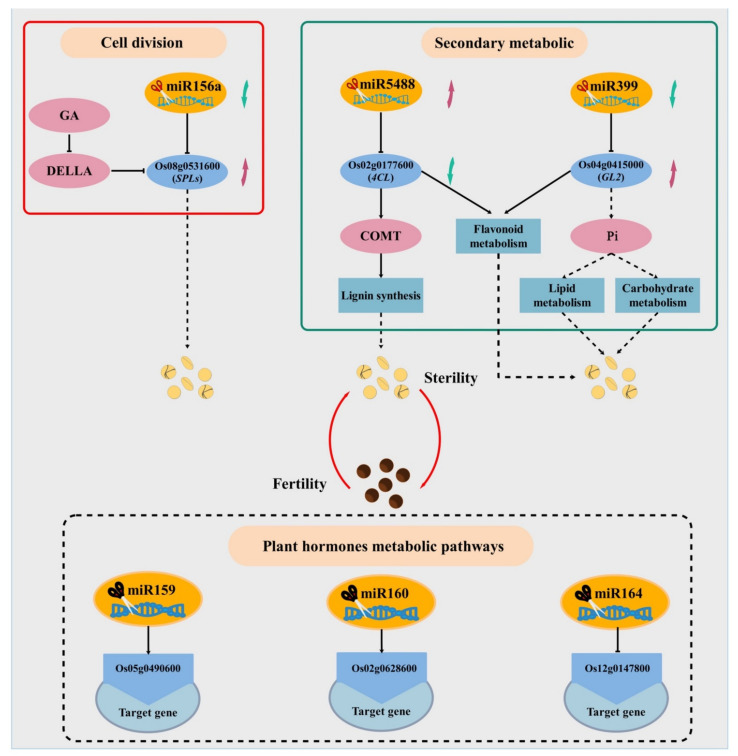
Model of miRNA and target gene regulation of male sterile changes in PA64S. The black dashed box indicates the miRNA and its target gene that did not reach a significant difference. The red arrow indicates the upregulated miRNA or target genes, and the green arrow indicates the downregulated miRNA or target genes.

Some miRNA targets such as miR159, miR160, miR164 and miR397 were not shown in the research results. Since these miRNAs did not reach a significant level of difference in bioinformatics analysis, from the perspective of differential expression, they still have a key role in rice fertility development. Some of these target genes are functionally classified as regulating plant hormone levels. However, auxin, gibberellin, cytokinin, and jasmonic acid play important roles in regulating plant sex differentiation, mediating the maturation of stamens and pollen, as well as the growth and development of plant vegetative and floral organs [57,92,93]. Therefore, we can infer that these insignificant miRNAs still have a certain regulatory role in PTGMS rice fertility changes, and we will pay attention to these insignificant miRNAs in subsequent studies. Finally, according to the comprehensive analysis, a possible model for miRNA to participate in the regulation of PTGMS rice fertility changes was established (Figure 11). We also verified our sequencing results by qPCR and found that both the identified miRNA and its target gene have a better expression trend in the sixth stage, indicating that compared to the seventh stage, the sixth stage is more suitable as sample selection for the analysis of fertility differences.

## 4. Materials and Methods

### 4.1. Plant Materials and Experimental Treatment

**Figure 12:**
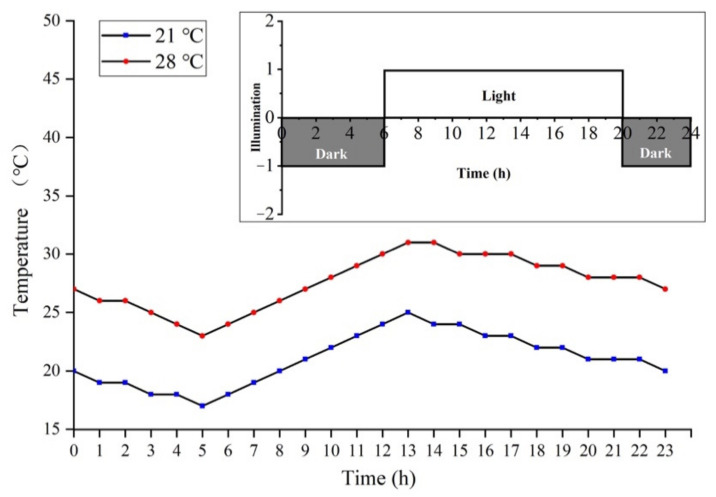
Temperature setting conditions for the low- and high-temperature treatment of PA64S. The red line represents the high-temperature treatment, and the blue line represents the low-temperature treatment. In the light intensity diagram, dark rectangles represent darkness, and light rectangles represent lights.

The PA64S used in this study was a PTGMS rice material that had been identified and bred by our research team for multiple generations. The critical temperature for male sterility is 23.0 °C. Under long light conditions, male sterility manifests when the average temperature is above 23.0 °C, and males are fertile at average temperatures below 23.0 °C. This experiment was conducted at the Crop Physiology and Production Centre of Huazhong Agricultural University (30.28 °N, 114.20 °E). Sowed annually on May 10, better growing seedlings were selected and transplanted into enamel pots to grow naturally. Three plants were transplanted into each pot, each limited to primary tillers. During this period, regular fertilization, watering, and disease management was performed. According to the method of the eight-stage differentiation of young rice spikes [94,95], when the plant population (50%) developed into the secondary stalk and spikelet primordium differentiation stage (stage 3), the rice plants were moved to the plant growth chamber. The average temperatures were 21 and 28 °C, with a fluorescent light of 300 μmol/(m^2^·s) and a relative humidity of 80%. The temperature changes and light conditions of each treatment are shown in Figure 12. After the pollen content filling stage (stage 7), all plants were moved to natural conditions until maturity, and the temperature change at the later growth stage is shown in Supplementary Figure S3. Thus, fertile PA64S(F) material at 21 °C and sterile PA64S(S) material at 28 °C were obtained. We selected the florets of PA64S(S) and PA64S(F) plants that were in the meiosis stage of pollen mother cells (stage 6) and the pollen content filling stage (stage 7). In each treatment, we selected 60 plants with more than two young spikelets per plant and then immediately froze them in liquid nitrogen for sequencing (small RNA, RNA, and degradome sequencing), qPCR validation, and metabolite content evaluation. We used S6 and F6 to represent PA64S(S) and PA64S(F) of stage 6, respectively, and S7 and F7 to represent PA64S(S) and PA64S(F) of stage 7, respectively.

### 4.2. Phenotype Analysis of PA64S

Photos of mature PA64S(F) and PA64S(S) rice plants were taken to observe the morphological differences. The anthers of the top floret that had initiated heading were picked and stained with 1% I_2_-KI. The anthers were extruded with tweezers to remove the residue, and three fields of view were observed randomly under the microscope. Pollen was classified into two categories—fertile and abortive (pollenless, round abortive, and spot abortive)—according to its different morphologies, and the percentage of fertility was calculated. Finally, the percent fertility of all tested spikelets from the same treatment was averaged to obtain the percentage of pollen fertility for the period under the treatment. The seed setting rate was calculated based on the statistics of the self-fruitfulness of different materials 25 days after heading. We selected 60 plants with more than two young spikelets per plant for observation, with pollen fertility and seed setting rate *N* > 120. Significant differences were determined according to Student’s *t*-test at *p* < 0.01 (**). At the same time, representative intact spikelets were selected and placed on a dissecting microscope to observe the difference in anther morphology.

### 4.3. Small RNA and RNA Sequencing

#### 4.3.1. Small RNA Sequencing and Data Analysis

Total RNA was isolated and purified using TRIzol reagent (Invitrogen, Carlsbad, CA, USA) following the manufacturer’s procedure. The RNA amount and purity of each sample were quantified using a NanoDrop ND-1000 (NanoDrop, Wilmington, DE, USA). RNA integrity was assessed by an Agilent 2100 with RIN number > 7.0. Approximately 1 μg of total RNA was used to prepare the sRNA library according to the TruSeq Small RNA Sample Prep Kit (Illumina, San Diego, CA, USA), and 50 bp single-end sequencing was performed on an Illumina HiSeq 2500 (LC Bio, Hangzhou, China) following the vendor’s recommended protocol. Each sample was represented by three biological replicates.

In this step, clean reads were obtained by removing reads containing poly-N, reads with 5′ adapter contaminants or without the 3′ insert adapter tag, reads containing poly A, T, G or C and reads low-quality reads from raw data. The small RNA tags were mapped to the reference sequence by Bowtie [96] without mismatch to analyze their expression and distribution on the reference. The clustering of the index-coded samples was performed on a cBot Cluster Generation System using TruSeq SR Cluster Kit v3-cBot-HS (Illumina) according to the manufacturer’s instructions. After cluster generation, the library preparations were sequenced on an Illumina HiSeq 2500/2000 platform, and 50 bp single-end reads were generated. Mapped small RNA tags were used to look for known miRNAs. miRBase20.0 was used as a reference, and the modified mirDeep2 [97] and sRNA-tools-cli software were used to obtain the potential miRNAs and draw the secondary structures. Custom scripts were used to obtain the miRNA counts and base bias on the first position of identified miRNA with a certain length and on each position of all identified miRNA. The available miREvo [98] and mirDeep2 software were integrated to predict novel miRNAs by exploring the secondary structure, the Dicer cleavage site, and the minimum free energy of the sRNA tags unannotated in the former steps. In our analysis pipeline, known miRNAs used miFam.dat (http://www.mirbase.org/ftp.shtml, accessed on 10 January 2021) to look for families; novel miRNA precursors were submitted to Rfam (http://rfam.xfam.org, accessed on 10 January 2021) to look for Rfam families. Predicting the target gene of miRNA was performed by PsRobot-tarin PsRobot [99] for plants.

#### 4.3.2. RNA Sequencing

Total RNA was extracted from PA64S (F) and PA64S (S) florets at the meiosis stage of pollen mother cells (stage 6) and the pollen content filling stage (stage 7) using the TIANGEN RNAprep Pure Plant Kit, as described by the supplier. For RNA sequencing, 3.0 μg of RNA from each sample was used for sequencing. Each sample was represented by three biological replicates.

RNA-Seq and bioinformatics analyses were conducted by Novogene (Beijing, China). Reference genome and gene model annotation files were directly downloaded from genome website. The reference genome information came from the Ensembl database (ftp://ftp.ensemblgenomes.org/pub/plants/current/fasta/oryza_indica, accessed on 10 January 2021). The URL for gene model annotation files is ftp://ftp.ensemblgenomes.org/pub/plants/current/gtf/oryza_indica, accessed on 10 January 2021. Sequencing libraries were generated according to the standard protocols provided by Illumina and sequenced on an Illumina HiSeq platform. Clean reads were obtained by removing reads containing adapters, reads containing poly-N, and low-quality reads from raw data. Then, clean reads were mapped to reference genome sequences of the Nipponbare (*japonica*) genome using Hisat2. The mapped reads of each sample were assembled by StringTie (v1.3.3b, CCB of Johns Hopkins University, Baltimore, USA). FeatureCounts (v1.5.0-p3, WEHI, Parkville Victoria, Australia) was used to count the read numbers mapped to each gene. Then, the FPKM of each gene was calculated based on the length of the gene and read count mapped to this gene. Gene differential expression analysis was performed using the DESeq2 R package. A corrected *p*-value of 0.05 and an absolute fold change of 2 were set as the threshold for significant differential expression. The GO enrichment analysis of differentially expressed genes was implemented by the clusterProfiler R package, in which gene length bias was corrected. After correction, GO items with a *p*-value of less than 0.05 were considered to be significantly enriched by differentially expressed genes.

### 4.4. Degradome Sequencing and Data Analysis

Poly(A) RNA was purified from plant total RNA (20 μg) using poly-T oligo-attached magnetic beads using two rounds of purification. Because the 3′ cleavage product of the mRNA contains a 5′-monophosphate, the 5’ adapters were ligated to the 5’ end of the 3′ cleavage product of the mRNA by RNA ligase. The next step is reverse transcription to make the first strand of cDNA with a 3′-adapter random primer, and size selection was performed with AMPureXP beads. Then, the cDNA was amplified with PCR under the following conditions: initial denaturation at 95 °C for 3 min; 15 cycles of denaturation at 98 °C for 15 s, annealing at 60 °C for 15 s, and extension at 72 °C for 30 s; and a final extension at 72 °C for 5 min. The average insert size for the final cDNA library was 200–400 bp. Finally, we performed 50 bp single-end sequencing on an Illumina HiSeq 2500 (LC Bio, China) following the vendor’s recommended protocol. Each sample was represented by three biological replicates.

Raw sequencing reads were obtained using Illumina’s software to remove adaptors and low-quality reads. The extracted sequencing reads were then used to identify potentially cleaved targets by the Cleveland pipeline [100]. The degradome reads were mapped to the databases. Only the perfect matching alignment(s) for the given read were kept for degradation analysis. All resulting reads (t-signature) were reverse-complemented and aligned to the miRNA identified in our study. No more than four alignment scores were allowed. Alignments where the degradome sequence position coincident with the tenth or eleventh nucleotide of miRNA were retained and scored. The target was selected and categorized as 0, 1, 2, 3 or 4. Category 0 was defined as having over one raw read at the position, abundance at the position that was equal to the maximum on the transcript, and only one maximum on the transcript. Category 1 was defined as having over one raw read at the position, abundance at the position that was equal to the maximum on the transcript, and more than one maximum position on the transcript. Category 2 was defined as having over one raw read at the position and abundance at position that was less than the maximum but higher than the median for the transcript. Category 3 was defined as having over one raw read at the position and abundance at the position that was equal to or less than the median for the transcript. Category 4 was defined as having only one raw read at the position. In addition, to easily analyze the miRNA targets and RNA degradation patterns, t-plots were built according to the distribution of signatures (and abundances) along these transcripts. All the identified targets were subjected to BlastX analysis to search for similarity and then to GO analysis to uncover the miRNA–gene regulatory network based on biological process, cellular component, and molecular function.

### 4.5. qPCR Validation

We randomly selected a total of six miRNAs for qPCR verification from miRNA sequencing, and six miRNA—target gene pairs were also selected for functional validation. Total RNA was extracted using the RNAprep Pure Plant Kit (TIANGEN, Beijing, China) according to the instructions. For mRNA quantification, we followed the instructions of the RevertAid^TM^ First Strand cDNA Synthesis Kit (MBI, Lithuania) to reverse-transcribe RNA samples into cDNA. In this experiment, *Acting7* (X16280) was selected as the internal reference gene. We used Primer 3.0 to design specific primers, and these primers were synthesized by Shanghai Shenggong Bioengineering Co., Ltd., Shanghai, China. The specific primer information is shown in Supplementary Table S8. The QuantStudio™ Real-Time PCR Detection System was used for qPCR, and each sample was represented by three biological replicates. For miRNA quantification, according to the instructions, we used a TIANGEN miRcute enhanced miRNA cDNA first-strand synthesis kit to obtain reverse transcription products in a PCR machine (Bio-Rad, Hercules, CA, USA). A TIANGEN miRcute Enhanced miRNA Fluorescence Quantitative Detection Kit (SYBR Green) was used for the qPCR of miRNA, and U6 sRNA was used as a reference gene for miRNA analysis. The expression of miRNA was verified by three biological replicates. Values are represented as the average values of three biological repeats. Error bars represent standard deviations. Asterisks indicate significant differences revealed by Student’s *t*-test at *p* < 0.05 (*).

### 4.6. Determination of Metabolite Content

#### 4.6.1. Flavonoids and Soluble Sugar Content

The flavonoid content was determined according to the work of Sumczynski [101] with a modification. Briefly, 20 mL of 70% ethanol were mixed with 5 mL of the extract and 0.5 mL of 5% NaNO_2_. After 6 min, 0.5 mL of 10% AlCl_3_·6H_2_O solution was added, and the mixture was allowed to stand for 6 min. Then, 4 mL of 4% NaOH were added. The absorbance was measured after 12 min at 510 nm.

Soluble sugar was determined according to the work of FU [102]. Frozen anthers (15 mg) were homogenized with deionized water, the extract was filtered, and the extract was treated with 5% phenol and 98% sulfuric acid. The mixture was kept for 1 h, and then absorbance at 485 nm was determined. The contents of soluble sugar were expressed as g·g^−1^ FW. Flavonoids and soluble sugar contents according to Student’s *t*-test at *p* < 0.01 (**), and each sample was represented by three biological replicates.

#### 4.6.2. Lipid Type and Content

Sixty milligrams of florets of PA64S (F) and PA64S (S) at stage 7 were used to dissolve the extracted lipids. A Nexera UPLC (Shimadzu, Kyoto, Japan) system fitted with a Q-Exactive quadrupole-orbitrap mass spectrometer equipped with a heated electrospray ionization (HESI) source (Thermo Fisher Scientific, Waltham, MA, USA) was used to analyze the lipidomic analyses. The acquired LCsingle bondMS raw data were analyzed by the progenesis QI software (version 2.3, Waters Corporation, Milford, MA, USA). The positive and negative data were combined to obtain combined data that were imported into the SIMCA software package (version 14.1, Umetrics, Umeå, Sweden). The VIP value of the first principal component of the OPLS-DA model was greater than 1, and the *p*-value of the *t* test was less than 0.05 as the criteria for screening differential lipids. Each sample was represented by five biological replicates.

## 5. Conclusions

The differential expression of miRNAs from different fertilities of PA64S indicates that miRNAs are involved in the development of anthers and male sterility in PTGMS rice. Through the integrated analysis of the miRNA–mRNA-degradation group, 13 pairs of miRNA–mRNA with significant differences that may be involved in the regulation of PA64S fertility changes were identified. We consider that miR156a, miR5488 and miR399d have prominent contributions in the regulation of fertility changes in rice. The results of these differentially expressed miRNAs and their targets in anthers provide a new understanding of PTGMS rice, which will help us better understand the potential regulatory mechanisms of male sterility in the future.

## Figures and Tables

**Table 1:** Pollen fertility and seed-setting rate of PA64S.

Year	Treatment	Pollen Fertility (%)	Seed Setting Rate (%)
2018	PA64S(S)	0.00	0.00
	PA64S(F)	41.35 ± 1.76 **	34.27 ± 5.99 **
2019	PA64S(S)	0.00	0.00
	PA64S(F)	30.72 ± 2.69 **	31.40 ± 7.87 **

Value are expressed as mean ± standard deviation, *n* > 120; Asterisks indicate significant differences revealed by Student’s *t*-test at *p* < 0.01 (**).

**Table 2:** Significantly differentially expressed miRNA families of PA64S.

miRNA Family	Stage 6			Stage 7		
	miRNA Number	Up	Down	miRNA Number	Up	Down
MIR156	1	1	0	-	-	-
MIR164	1	0	1	-	-	-
MIR396_2	1	1	0	-	-	-
MIR397	2	0	2	-	-	-
MIR5079	1	1	0	-	-	-
MIR5143	1	1	0	-	-	-
MIR171_1	1	1	0	1	1	0
MIR1861	2	2	0	4	4	0
MIR1862	1	1	0	2	2	0
MIR1863	1	1	0	1	1	0
MIR1878	1	1	0	1	1	0
MIR2118	2	0	2	1	1	0
MIR2863	1	1	1	1	1	0
MIR437	1	1	0	1	1	0
MIR812	3	1	2	2	1	1
MIR169_1	-	-	-	1	1	0
MIR169_2	-	-	-	2	2	0
MIR169_4	-	-	-	1	1	0
MIR1883	-	-	-	1	1	0
MIR2275	-	-	-	2	1	1
MIR395	-	-	-	4	4	0
MIR399	-	-	-	3	3	0
MIR444	-	-	-	1	0	1
MIR5160	-	-	-	1	0	1
MIR529	-	-	-	1	0	1
MIR820	-	-	-	2	0	2
MIR827	-	-	-	1	1	0

**Table 3:** Categories of candidate cleaved sites by degradome sequencing.

Degradome Category Type	Cleavages Events	Genes	miRNAs
Category 0	633	287	89
Category 1	99	68	59
Category 2	1440	960	262
Category 3	195	161	75
Category 4	851	776	233

**Table 4:** The regulatory miRNA–mRNA interaction pairs in PA64S(S) compared with PA64S(F).

miRNA	Target Genes	Relative Expression Level in Response to Fertility				Alignment Range	Cleavage Site	Category	
		miRNA log2	miRNA	Target log2	Target			
osa-miR156a	OS08G0531600	−1.81	down	2.04	up	1053–1073	1064	2
osa-miR164a	OS06G0675600	0.32	up	−2.58	down	954–974	965	0
osa-miR528-5p	OS09G0365900	1.05	up	−2.41	down	164–183	174	2
	OS12G0552300	1.05	up	1.83	up	2790–2809	2800	4
osa-miR5488	OS02G0177600	1.22	up	−2.14	down	1516–1535	1526	2
osa-miR171b	OS10G0551200	−0.83	down	−1.16	down	549–569	560	2
	OS05G0417100	−0.83	down	0.87	up	1957–1977	1968	2
osa-miR319a-3p.2-3p	OS01G0755500	−0.97	down	−1.09	down	1236–1254	1245	0
	OS03G0785800	−0.97	down	−1.14	down	1182–1201	1192	0
osa-miR396c-5p	OS04G0600900	0.52	up	−1.78	down	404–425	415	0
	OS02G0776900	0.52	up	−1.26	down	570–590	581	0
osa-miR156l-5p	OS01G0922600	−1.81	down	−0.70	down	615–635	626	0
osa-miR172d-5p	OS02G0582400	1.21	up	−0.75	down	671–689	681	2
osa-miR399a	OS04G0415000	−1.36	down	0.90	up	489–510	500	2
osa-miR399d	OS04G0415000	−1.14	down	0.90	up	489–510	500	2
osa-miR399j	OS04G0415000	−1.10	down	0.90	up	489–510	500	2
osa-miR419	OS01G0606000	−1.86	down	2.44	up	108–127	119	4

## Supplementary Materials

The following are available online at https://www.mdpi.com/1422-0067/22/5/2260/s1, Figure S1: Correlation analysis of the sample in the sequencing libraries. Figure S2: Target plots showing signature abundance in the position of identified target transcripts. Figure S3: Trend of natural temperature variation in the growing season. Table S1: The statistics of sRNA-seq reads in stage 6 and stage 7 of PA64S(S) and PA64S(F). Table S2: The statistical of sRNA mapped in stage 6 and stage 7 of PA64S(S) and PA64S(F). Table S3: The results of miRNAs identified in stage 6 and stage 7 of PA64S(S) and PA64S(F). Table S4: The statistics of mRNA-seq reads and mapped reads in stage 6 and stage 7 of PA64S(S) and PA64S(F). Table S5: The results of DEGs in stage 6 and stage 7 of PA64S(S) and PA64S(F). Table S6: The statistics degradome-seq reads in stage 6 and stage 7 of PA64S(S) and PA64S(F). Table S7: The results of the targets identified with degradome analysis in stage 6 and stage 7 of PA64S(S) and PA64S(F). Table S8: Primer information for miRNAs and target genes. Supplementary file 1: Secondary structure figure of novel miRNAs.
