# Supplementary material for: Integrated Analysis of Small RNA, Transcriptome, and Degradome Sequencing Reveals the MiR156, MiR5488 and MiR399 Are Involved in the Regulation of Male Sterility in PTGMS Rice
Source: Int J Mol Sci. 2021 Feb 24;22(5):2260. doi: 10.3390/ijms22052260 (PMC7956645; doi:10.3390/ijms22052260)
Supplement: Supplementary file 1 [file ijms-22-02260-s001.zip › ijms-1089447-proofreading done-supp/supplementary/Supplementary figure S1-S3.docx]

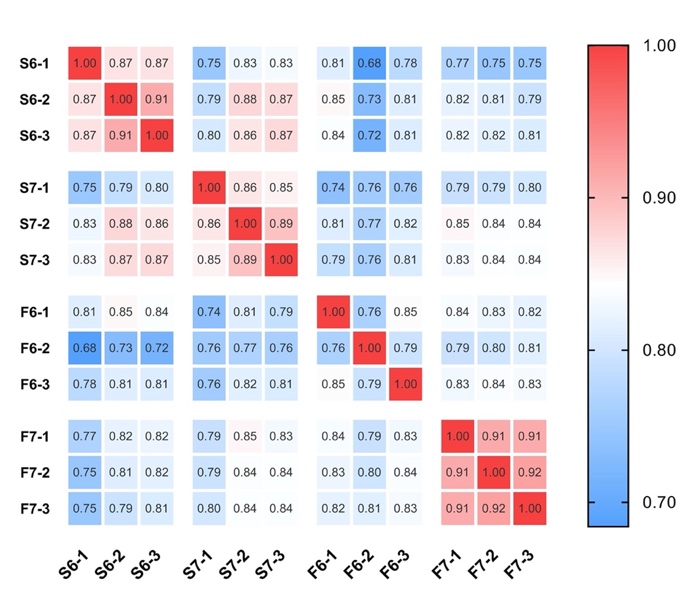


**Figure S1.** Correlation analysis of the sample in the sequencing libraries.


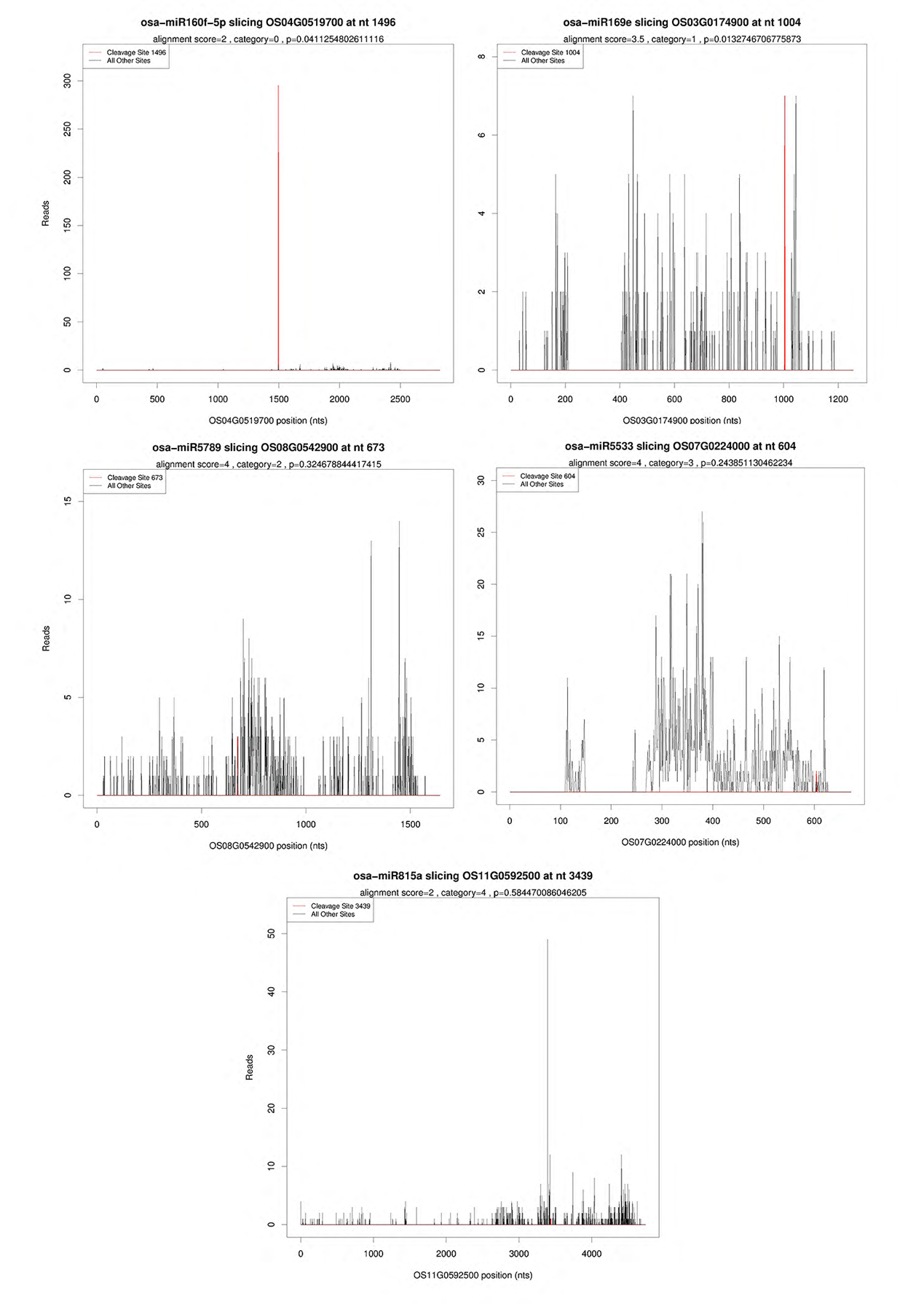


**Figure S2.** Target plots showing signature abundance in the position of identified target transcripts.


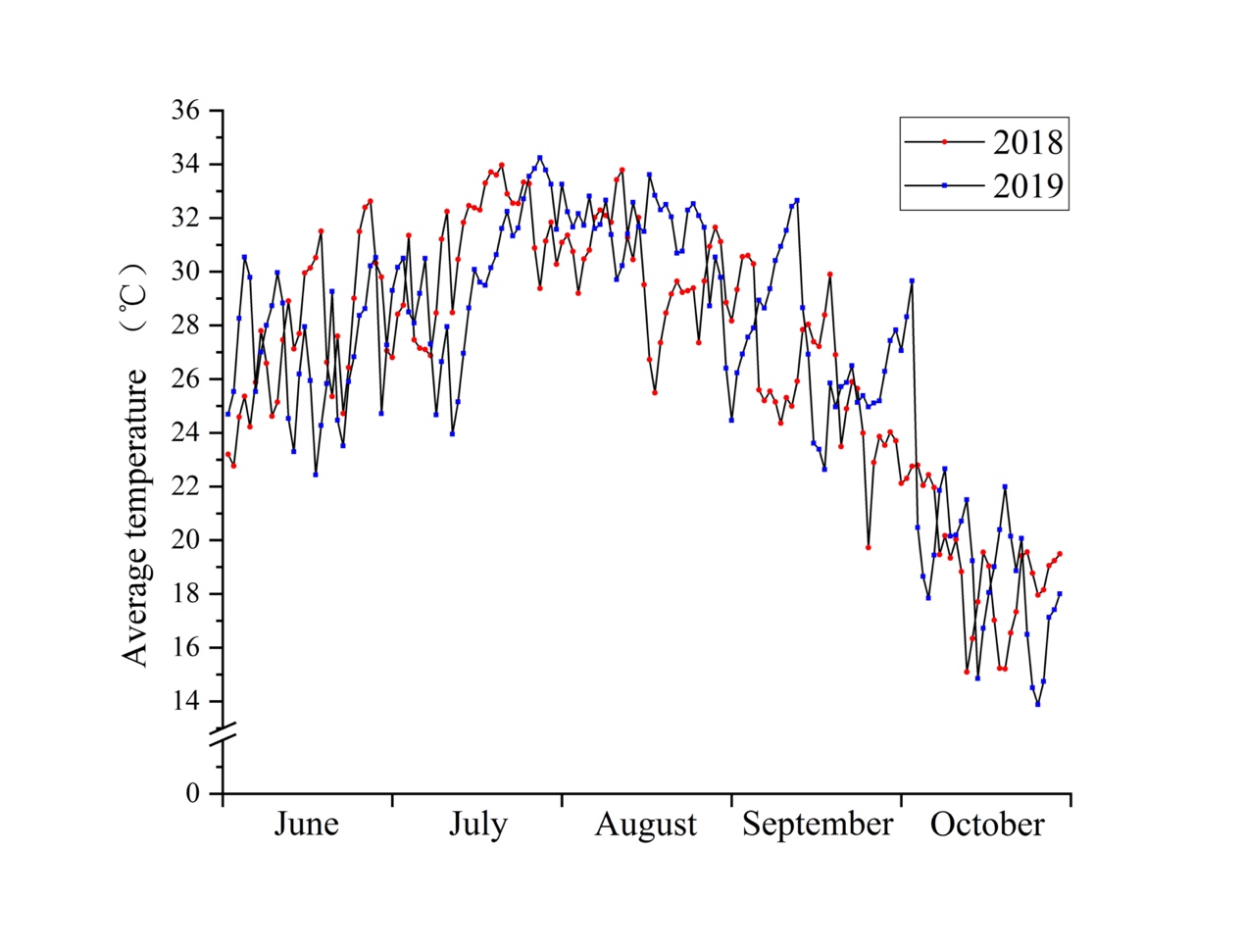


**Figure S3.** Trend of natural temperature variation in the growing season.
