# Supplementary figures and images for: Integrated Analysis of Small RNA, Transcriptome, and Degradome Sequencing Reveals the MiR156, MiR5488 and MiR399 Are Involved in the Regulation of Male Sterility in PTGMS Rice
Source: Int J Mol Sci. 2021 Feb 24;22(5):2260. doi: 10.3390/ijms22052260 (PMC7956645; doi:10.3390/ijms22052260)

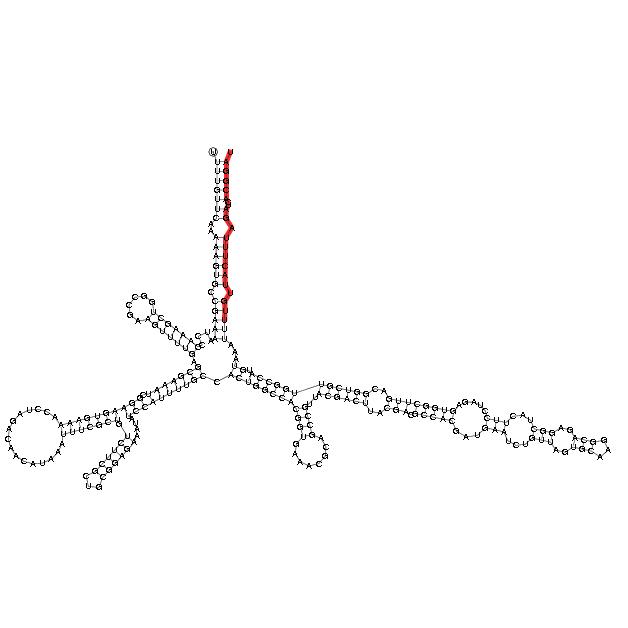

Supplement: Supplementary file 1 [file ijms-22-02260-s001.zip › ijms-1089447-proofreading done-supp/supplementary/Supplementary file 1/novel_100_novel_100.jpg]

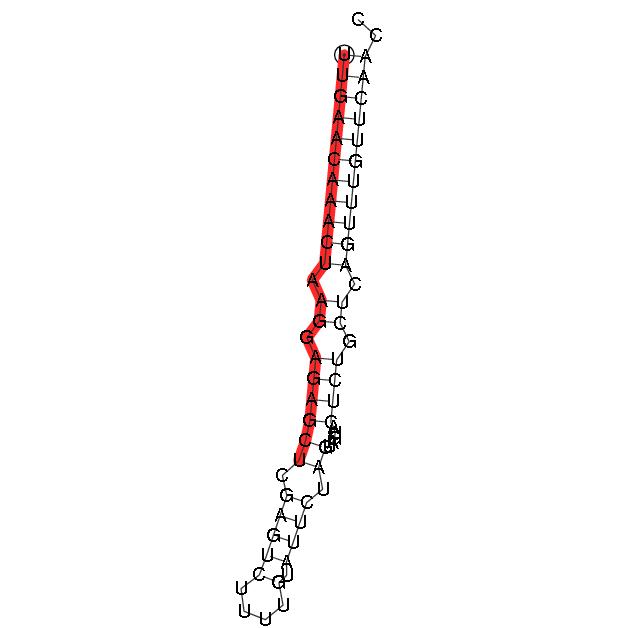

Supplement: Supplementary file 1 [file ijms-22-02260-s001.zip › ijms-1089447-proofreading done-supp/supplementary/Supplementary file 1/novel_110_novel_110.jpg]

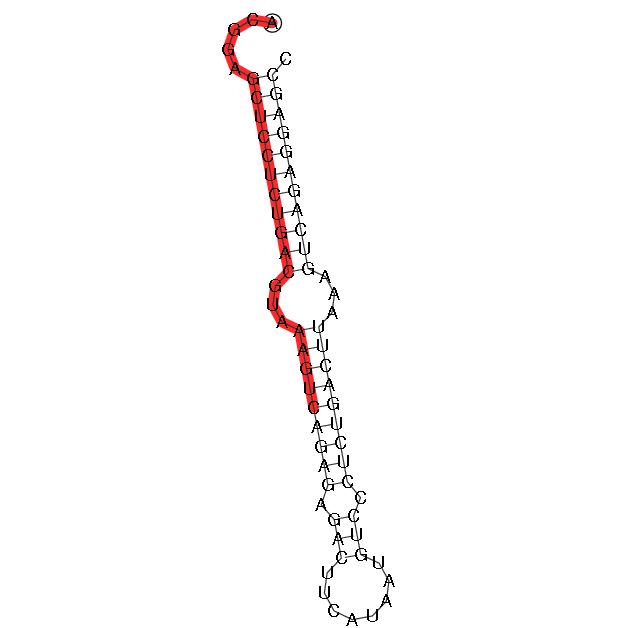

Supplement: Supplementary file 1 [file ijms-22-02260-s001.zip › ijms-1089447-proofreading done-supp/supplementary/Supplementary file 1/novel_114_novel_114.jpg]

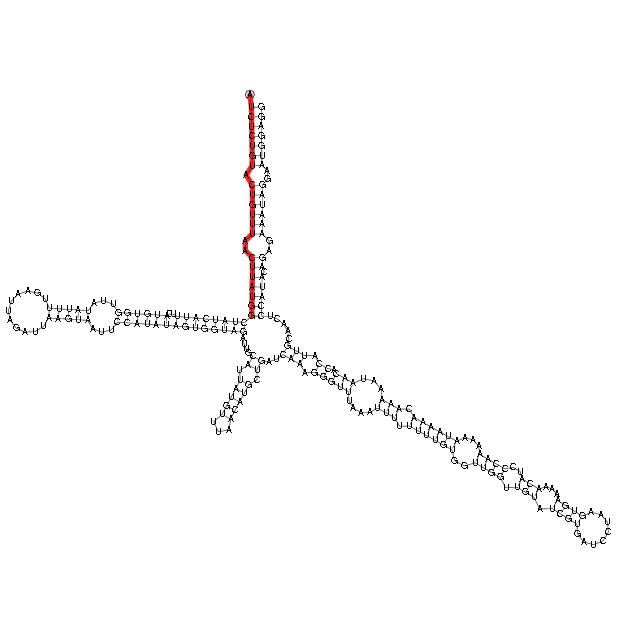

Supplement: Supplementary file 1 [file ijms-22-02260-s001.zip › ijms-1089447-proofreading done-supp/supplementary/Supplementary file 1/novel_121_novel_121.jpg]

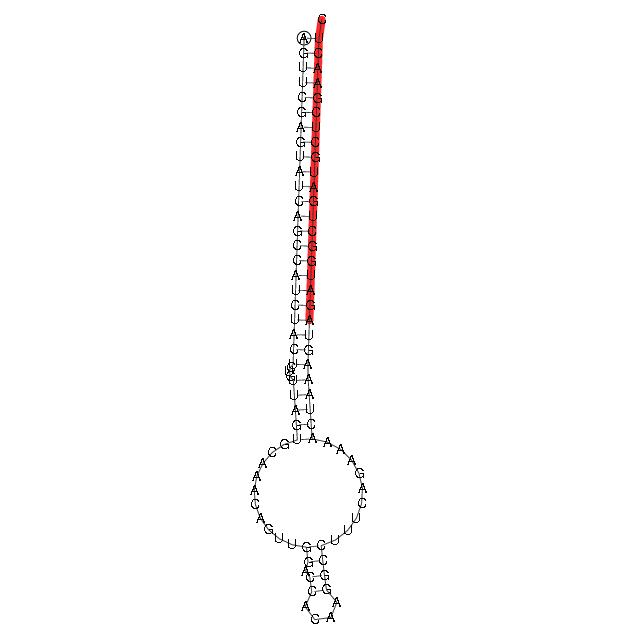

Supplement: Supplementary file 1 [file ijms-22-02260-s001.zip › ijms-1089447-proofreading done-supp/supplementary/Supplementary file 1/novel_138_novel_138.jpg]

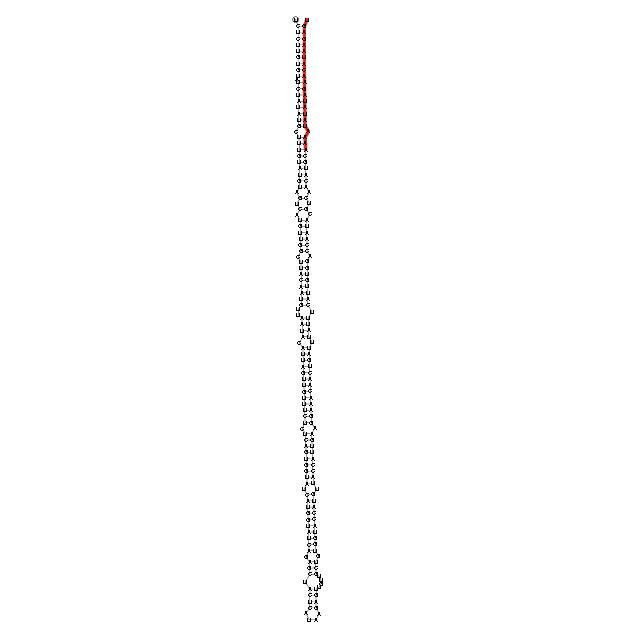

Supplement: Supplementary file 1 [file ijms-22-02260-s001.zip › ijms-1089447-proofreading done-supp/supplementary/Supplementary file 1/novel_189_novel_189.jpg]

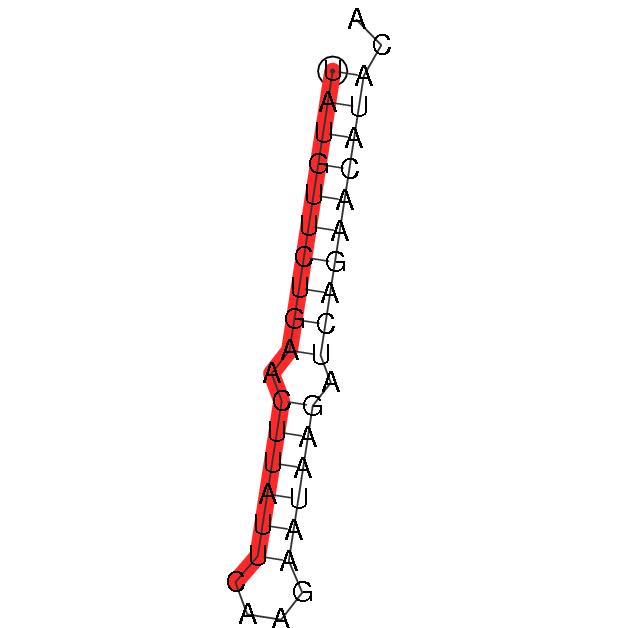

Supplement: Supplementary file 1 [file ijms-22-02260-s001.zip › ijms-1089447-proofreading done-supp/supplementary/Supplementary file 1/novel_198_novel_198.jpg]

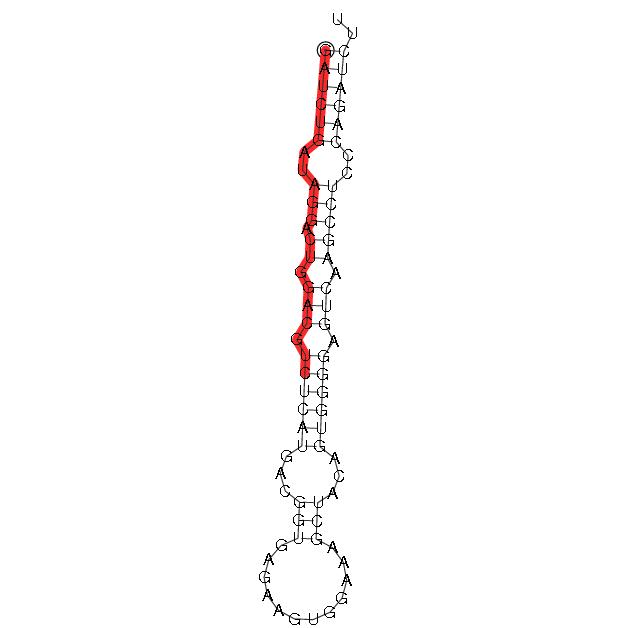

Supplement: Supplementary file 1 [file ijms-22-02260-s001.zip › ijms-1089447-proofreading done-supp/supplementary/Supplementary file 1/novel_215_novel_215.jpg]

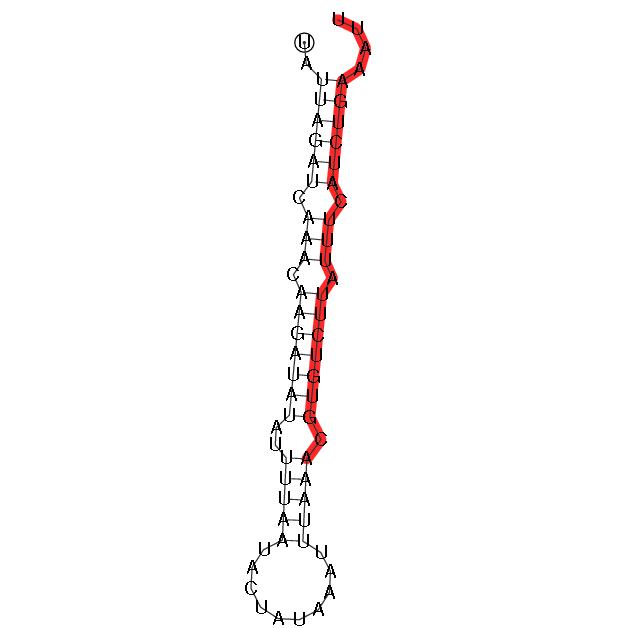

Supplement: Supplementary file 1 [file ijms-22-02260-s001.zip › ijms-1089447-proofreading done-supp/supplementary/Supplementary file 1/novel_230_novel_230.jpg]

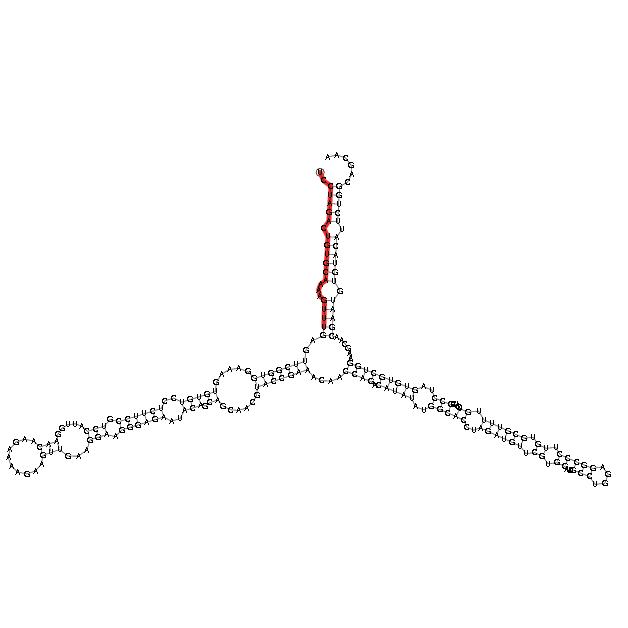

Supplement: Supplementary file 1 [file ijms-22-02260-s001.zip › ijms-1089447-proofreading done-supp/supplementary/Supplementary file 1/novel_30_novel_30.jpg]

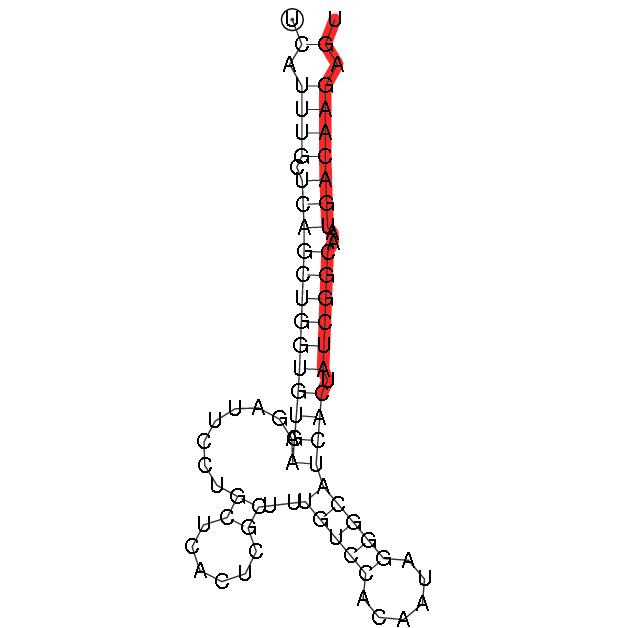

Supplement: Supplementary file 1 [file ijms-22-02260-s001.zip › ijms-1089447-proofreading done-supp/supplementary/Supplementary file 1/novel_34_novel_34.jpg]

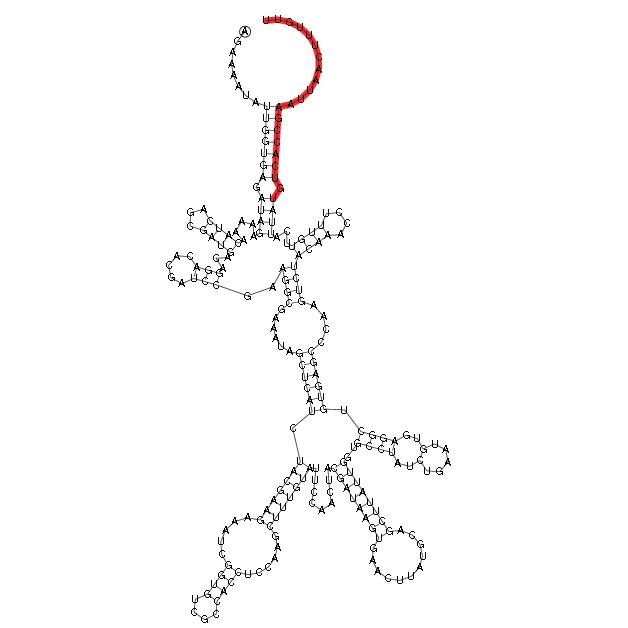

Supplement: Supplementary file 1 [file ijms-22-02260-s001.zip › ijms-1089447-proofreading done-supp/supplementary/Supplementary file 1/novel_39_novel_39.jpg]

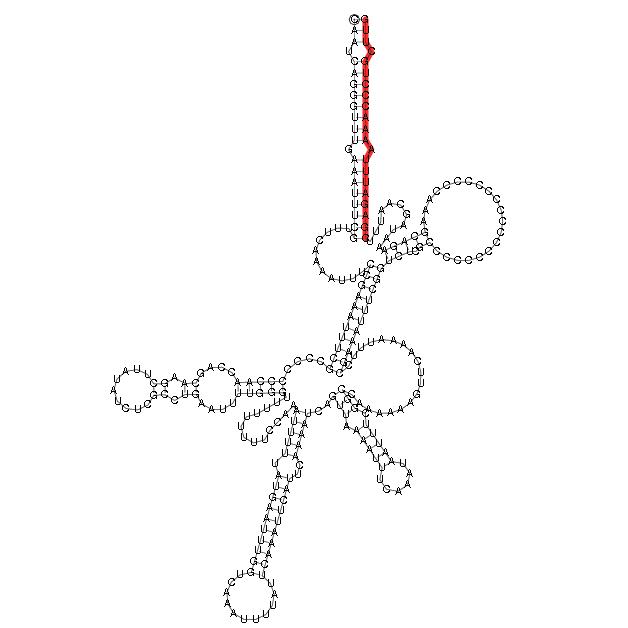

Supplement: Supplementary file 1 [file ijms-22-02260-s001.zip › ijms-1089447-proofreading done-supp/supplementary/Supplementary file 1/novel_41_novel_41.jpg]

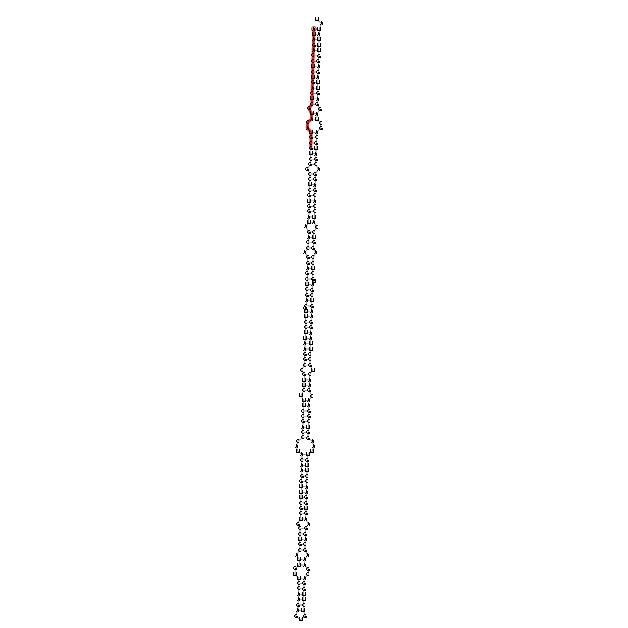

Supplement: Supplementary file 1 [file ijms-22-02260-s001.zip › ijms-1089447-proofreading done-supp/supplementary/Supplementary file 1/novel_42_novel_42.jpg]

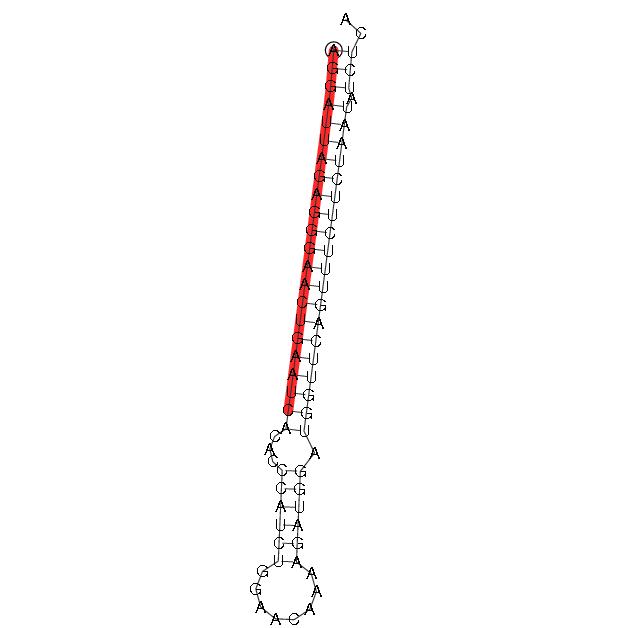

Supplement: Supplementary file 1 [file ijms-22-02260-s001.zip › ijms-1089447-proofreading done-supp/supplementary/Supplementary file 1/novel_46_novel_46.jpg]

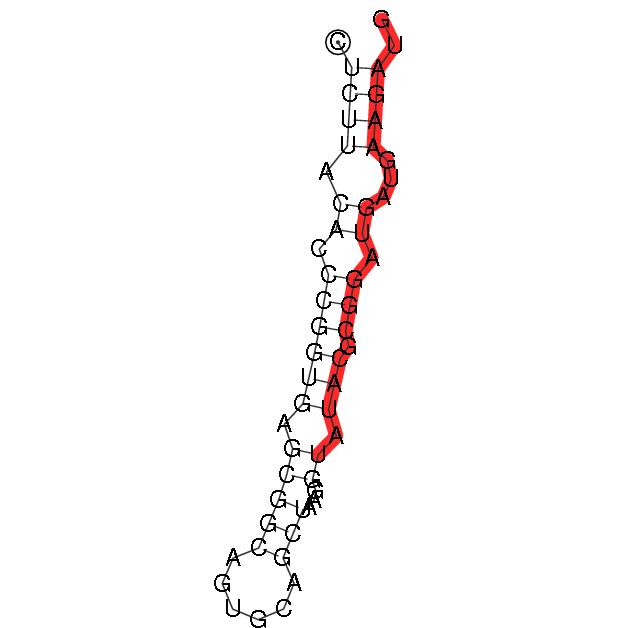

Supplement: Supplementary file 1 [file ijms-22-02260-s001.zip › ijms-1089447-proofreading done-supp/supplementary/Supplementary file 1/novel_60_novel_60.jpg]

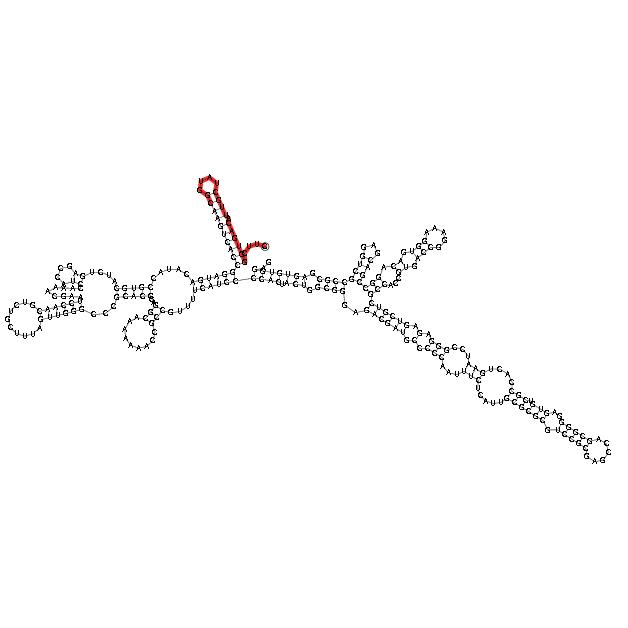

Supplement: Supplementary file 1 [file ijms-22-02260-s001.zip › ijms-1089447-proofreading done-supp/supplementary/Supplementary file 1/novel_64_novel_64.jpg]

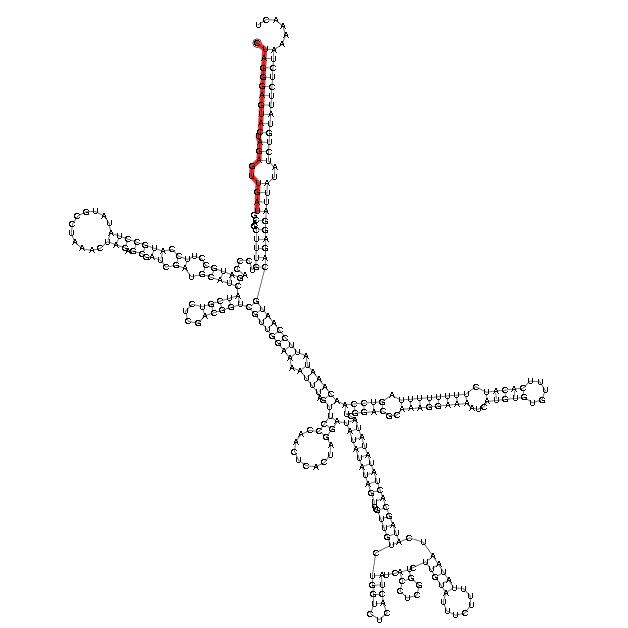

Supplement: Supplementary file 1 [file ijms-22-02260-s001.zip › ijms-1089447-proofreading done-supp/supplementary/Supplementary file 1/novel_85_novel_85.jpg]

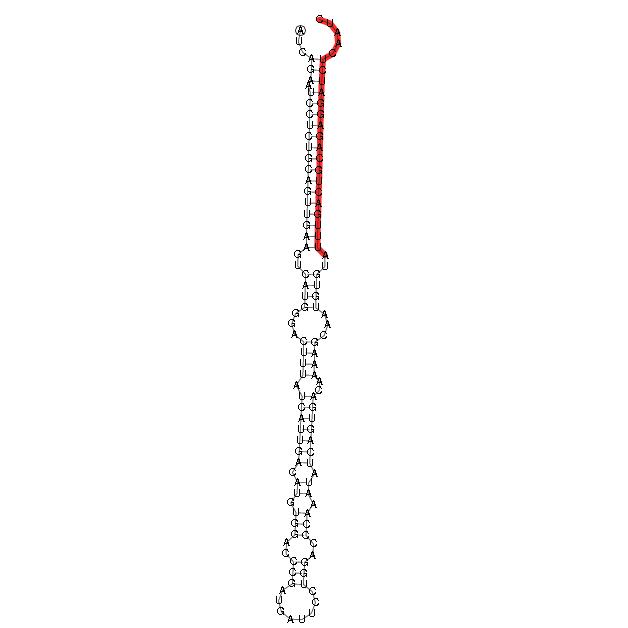

Supplement: Supplementary file 1 [file ijms-22-02260-s001.zip › ijms-1089447-proofreading done-supp/supplementary/Supplementary file 1/novel_89_novel_89.jpg]

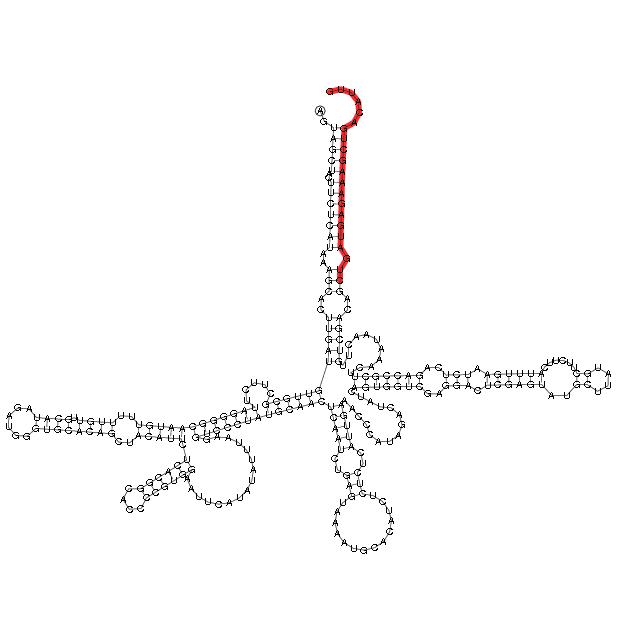

Supplement: Supplementary file 1 [file ijms-22-02260-s001.zip › ijms-1089447-proofreading done-supp/supplementary/Supplementary file 1/novel_92_novel_92.jpg]
